# Revisiting bacterial spore germination in the presence of peptidoglycan fragments

**DOI:** 10.1128/jb.00146-25

**Published:** 2025-07-03

**Authors:** Rosa Heydenreich, Juliana Nacita, Chia-Wei Lin, Finn O’Dea, Stéphane Mesnage, Graham Christie, Alexander Mathys

**Affiliations:** 1 Sustainable Food Processing Laboratory, Institute of Food, Nutrition, and Health, Department of Health Science and Technology, ETH Zurich 31089 , Zurich, Switzerland; 2 Department of Chemical Engineering and Biotechnology, University of Cambridge 151957 , Cambridge, United Kingdom; 3 Functional Genomics Center Zurich, ETH Zurich 27219, Zurich, Switzerland; 4 School of Biosciences, Molecular Microbiology, University of Sheffield 152813, Sheffield, United Kingdom; The Ohio State University, Columbus, Ohio, USA

**Keywords:** *Bacillus*, spores, germination, peptidoglycan, muropeptides

## Abstract

Bacterial spores of *Bacillus* species are metabolically inert cell types formed in response to nutrient starvation. Spores must undergo the process of germination to resume vegetative growth. This process is stimulated by the interaction of various nutrient molecules with specialized clusters of membrane-localized germinant receptors (GRs) present within spores. A second route to spore germination involving the stimulation of the PrkC Ser/Thr kinase by soluble peptidoglycan fragments was proposed in 2008 and has been subject to much less scrutiny. The current study examined the germinative response of spores of *Bacillus subtilis*, *Bacillus cereus,* and *Bacillus megaterium* when incubated in the presence of complex mixtures of peptidoglycan fragments or purified peptidoglycan fragments previously identified as germinants. The spore suspensions did not show any appreciable germination, as determined by fluorometric dipicolinic acid release, flow cytometry, or microscopy. However, the purified peptidoglycan fragments displayed a stimulatory effect on germination triggered by amino acids and nucleosides with spore GRs. In contrast, GR-mediated germination was inhibited to varying degrees by unidentified components of the complex peptidoglycan fragment mixtures derived from enzymatic digests of *B. subtilis* vegetative sacculi. Collectively, our results indicate that soluble peptidoglycan fragments cannot initiate spore germination but may influence germination via mechanisms that have yet to be established.

**IMPORTANCE:**

Stimuli and mechanisms that underpin bacterial spore germination are fairly well characterized. The physiological route relies upon the interaction of various small nutrient molecules with receptor proteins buried within spores. An alternative route to germination, whereby fragments of bacterial cell wall material—peptidoglycan—were proposed to stimulate a different receptor system, was proposed more recently (I. M. Shah, M. H. Laaberki, D. L. Popham and J. Dworkin, Cell 135:486–496, 2008, https://doi.org/10.1016/j.cell.2008.08.039). Results from the current study, where spores of several species of *Bacillus* were exposed to various peptidoglycan fragment-containing solutions, do not support this model of germination. This is significant since knowledge of germination can be exploited in a practical sense, as germinated spores are much easier to eradicate—in food processing and healthcare settings, for example—than their dormant counterparts.

## INTRODUCTION

Certain members of the family Bacillaceae, including *Bacillus* and related genera, can form endospores in response to nutrient starvation. The resultant spores are morphologically and physiologically distinct from their vegetative cellular counterparts, comprising a series of unique structural features and chemical signatures that render the spore metabolically dormant and resistant to numerous physicochemical and biological stress factors (1). Despite their inert nature, spores retain the ability to respond remarkably quickly to environmental cues that signal conditions conducive to vegetative growth (2). Receptor complexes that are cognate for nutrient-type molecules—typically amino acids and monosaccharides—are localized to the spore inner membrane and function as ligand-gated ion channels (3). The release of cations from these germinant receptor (GR) complexes when stimulated by appropriate ligands (germinants) represents the earliest event in the germination cascade that ultimately results in the emergence of a new vegetative cell.

**Fig 1:**
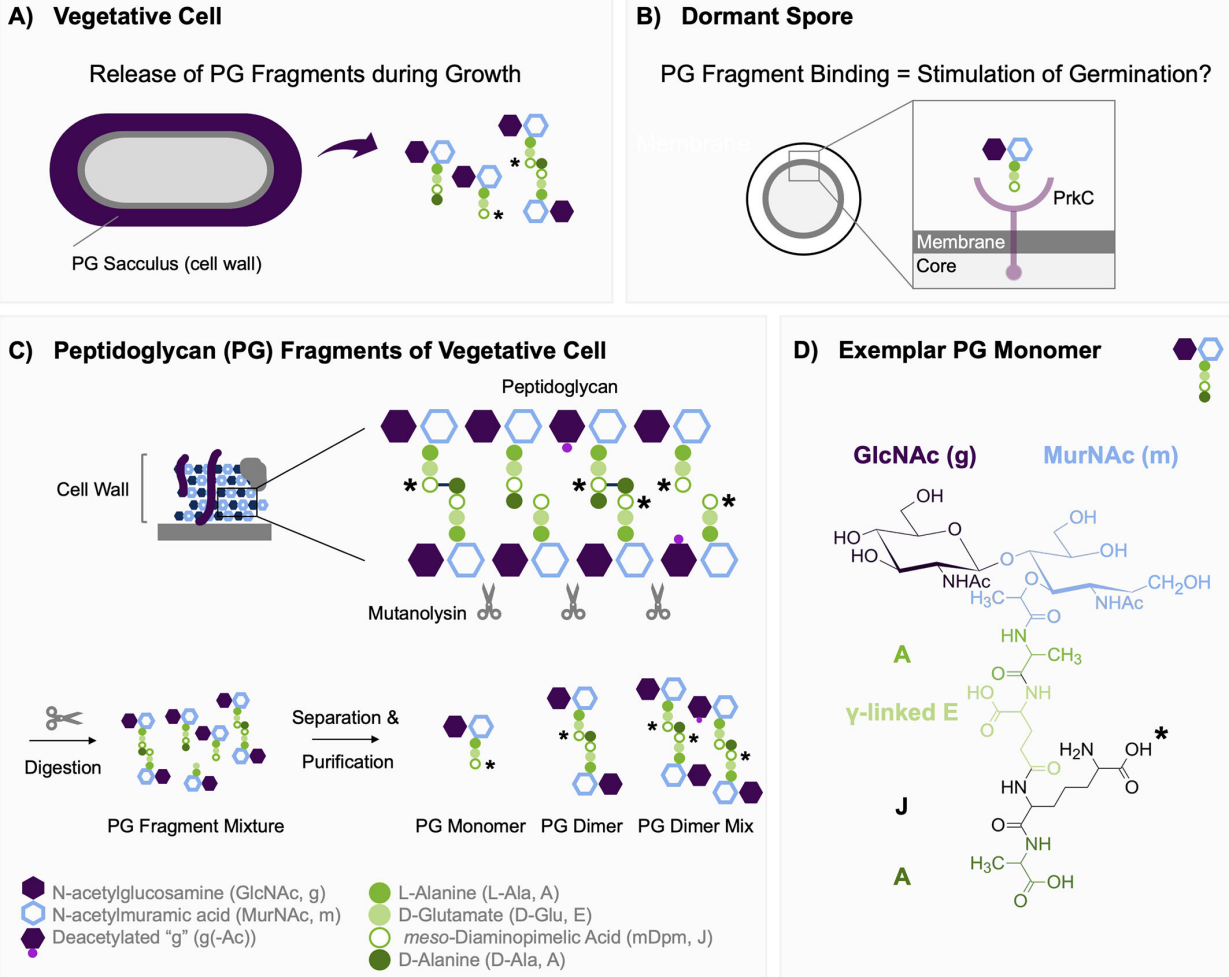
(A) Bacterial cells release peptidoglycan (PG) fragments during growth, which (B) were proposed to stimulate germination of dormant *Bacillus* spores (1). (C) *B. subtilis* PG is composed of linear chains of disaccharide peptide subunits that are crosslinked to varying degrees via peptide meso-diaminopimelic acid (mDpm) and D-alanine moieties. Mutanolysin, an N-acetylmuramidase, can enzymatically cleave the bacterial PG sacculus to generate a mixture of PG fragments of varying size and identity (see scissors). (D) In PG fragments with amidated mDpm, the OH group of the free COOH group (*) is replaced by NH_2_.

A second physiological route to germination, whereby spores exposed to certain peptidoglycan (PG) fragments, such as those released into the culture medium by actively growing populations of bacterial cells, are stimulated to germinate, was postulated more recently (Fig. 1) (4). In this scenario, an inner membrane-localized serine/threonine kinase named PrkC binds PG fragments and subsequently phosphorylates the core-localized ribosomal elongation factor EF-G. Conceivably, as speculated by the authors of the study (4), activated EF-G could contribute to the initiation of ribosomal translation of residual mRNA transcripts present in dormant spores, which somehow results in a germination process that at some point converges with the GR-mediated process. In the interim period, molecular details concerning PrkC recognition and binding of defined muropeptides have emerged (5, 6), but crucially not how phosphorylated EF-G or any PrkC-related downstream signaling event contributes to spore germination. Indeed, if we exclude work tangentially related to the original study, only one independent study demonstrating PG-mediated spore germination has been published over the same period (7). Perhaps significantly, as we’ll see below, that particular study employed a PG fragment mixture from enzymatically digested PG rather than purified PG fragments to stimulate germination of *Bacillus subtilis* spores with and without accompanying high hydrostatic pressure (HHP).

The present study was similarly initiated in part by an HHP-related project concerned with potential synergism between germinants and spore inactivation procedures, and also by reservations concerning the signal transduction scheme postulated for PG fragment-stimulated germination. Kinase activity, for example, requires a source of ATP, where there are only vanishingly small amounts—if any—in dormant spores (8, 9). Macromolecular mobility within the spore core, such as that presumably required by the initiation factors necessary for the assembly of functional ribosomes, is extremely constrained within this highly mineralized cellular compartment (10). Residual mRNA transcripts are also scarce and often fragmented, and it remains doubtful that they encode proteins that are relevant to the initiation or even early stages of germination (11). Likewise, the core of dormant spores is notable for containing few free amino acids at significant abundance, while the associated biosynthetic enzymes are largely absent (12). Similar kinetic and thermodynamic concerns can be applied to EF-G and other components—tRNA molecules, EF-Tu, GTP, etc.—that are required for any degree of protein translation in spores. Equally, it might not be a requirement for PrkC-mediated signal transduction to extend through to translation to initiate germination, and with spores, it is perhaps dangerous to assume that conventional physiological rules always apply (13). Thus, while EF-G-GTP is essential for ribosomal translocation, it is feasible that in this context, activated EF-G represents a secondary or downstream product of PrkC activity and does not necessarily represent the signal transduction event associated with the initiation of germination. In keeping with GR-mediated germination, germination initiation would presumably involve PrkC-mediated outflow of cations from the spore core, although it is not immediately obvious how this might be achieved.

Regardless of these hypothetical scenarios, the results of the current work do not support the hypothesis that PG fragments can initiate spore germination. Instead, we provide evidence that non-PG components present in the spent culture medium and in the complex PG fragment mixtures are probably responsible for triggering germination, and this is effected via the GRs. Indeed, unidentified component(s) of the latter can exert inhibitory effects on certain GR-mediated pathways. In a similar vein, purified PG fragments were not observed to trigger germination in spores of any of the strains and species tested in this work, but we demonstrate that they may exert a positive downstream effect on germination triggered via the GR pathway.

## RESULTS

### Spore germination in spent culture medium

**Fig 2:**
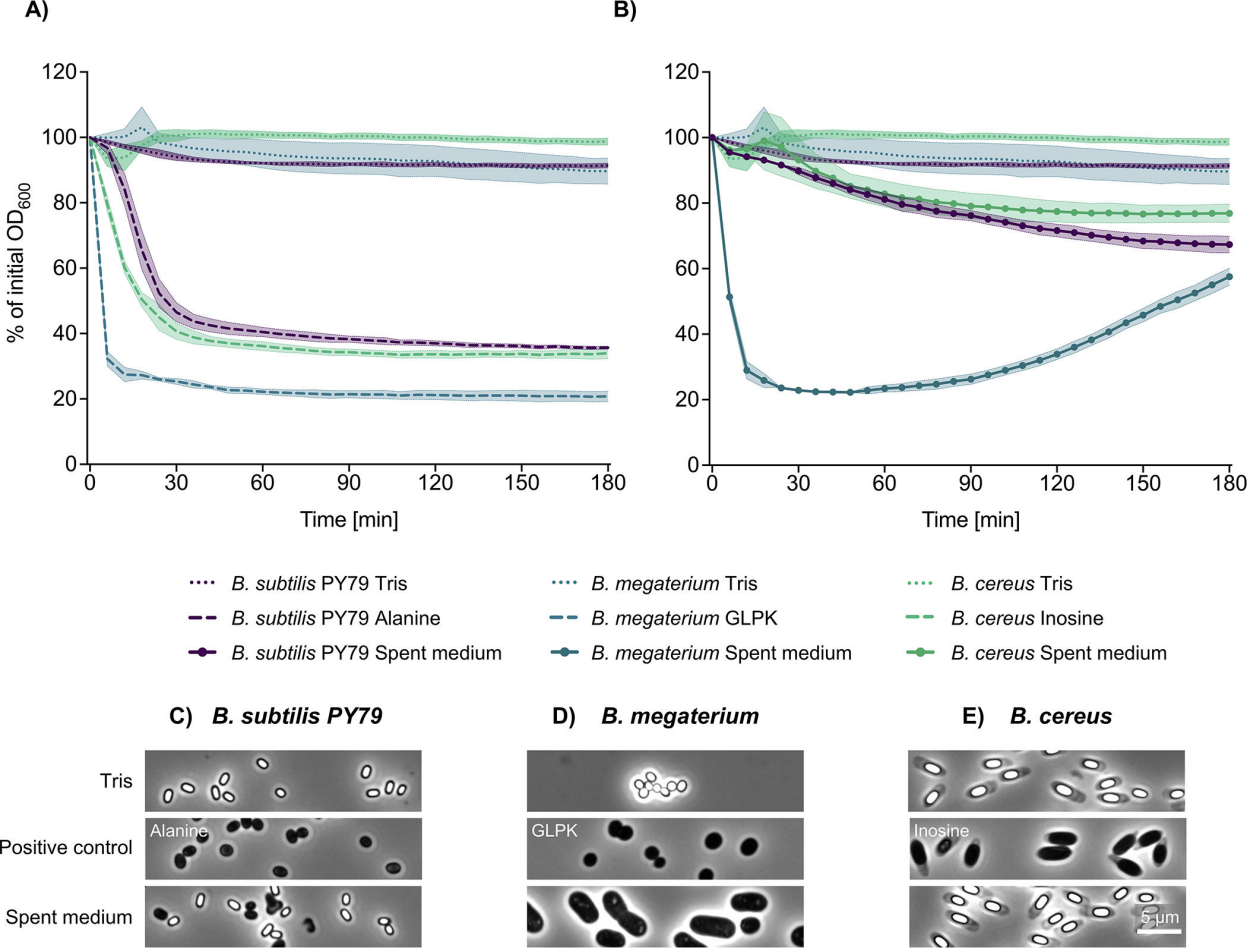
Germination of *B. subtilis* PY79, *B. megaterium,* and *B. cereus* spores as measured by changes in absorbance and phase contrast microscopy with time. Heat-shocked spores were incubated for 3 h at 37°C in (**A**) 10 mM Tris-HCl, pH 8 with or without nutrient germinants (10 mM L-alanine for *B. subtilis*, 10 mM inosine for *B. cereus*, or 10 mM each of D-glucose, L-leucine, and L-proline plus 50 mM KBr (GLPK) for *B. megaterium*), or (**B**) *B. subtilis* PY79 spent medium. Data are presented as the mean of technical triplicates from two independent biological spore replicates with error envelopes representing the standard deviation. (**C through E**) Representative phase contrast images of indicated spores post-incubation in spent medium or germination buffers. The scale bar of 5 µm applies to all images.

The initial idea that spore germination could be triggered by PG fragments arose from the observation that *B. subtilis* PY79 spores appeared to lose heat resistance when incubated in spent minimal medium (4), i.e., the cell-free supernatant of a bacterial culture depleted in nutrients. Loss of heat resistance is an early event in spore germination, presumably associated with the influx of water to the core at or around the same time that divalent and calcium dipicolinic acid (DPA) efflux occurs and is measured by plate-count assays following exposure of spore populations to appropriate thermal treatments. Since the minimal medium in question was not supplemented with free amino acids other than tryptophan, nor any other nutrient-type molecules that individually activate spore GRs, spore germination was determined to have been triggered by PG fragments shed into the spent culture medium during prior bacterial growth (4). We sought to repeat these experiments using the same *B. subtilis* PY79 strain and spent minimal medium as employed in the initial study. We additionally tested spores of two other species, *Bacillus megaterium* QM B1551 and *Bacillus cereus* 10876, for their germinative response in spent medium, since spores of the former species and a closely related member of the latter (*B. anthracis*) were demonstrated to germinate under these conditions previously (4). As a control measure, spores of all three species were demonstrated to germinate normally via their respective GR-mediated routes as determined from absorbance and phase contrast microscopy analyses (Fig. 2; note that the phosphate content of minimal medium precluded the use of terbium fluorescence as an assay for DPA release in these experiments [Supplemental S1]).

Spores of *B. subtilis* PY79 appeared to germinate over a 3 hour period in spent medium, albeit with much less efficiency than with saturating concentrations of defined nutrient germinants, whereas absorbance measurements for *B. megaterium* with spent medium were comparable with the defined germinant mixture (Fig. 2A and B). The marked increase in absorbance following germination of *B. megaterium* spores is indicative of population growth, which was verified subsequently by microscopy, which revealed the presence of vegetative cells undergoing binary fission (Fig. 2D). Evidently, the spent medium—perhaps supplemented by spore components generated and/or released during germination—contained sufficient nutrients to sustain a degree of population growth. Similar microscopy analyses of *B. subtilis* spores after 3 hours in spent medium revealed a mixture of phase-bright dormant and phase-dark germinated spores, some of which had evidently progressed to outgrowth (Fig. 2C). Surprisingly, only phase-bright dormant spores of *B. cereus* were observed after 3 hours in spent medium (Fig. 2E). The latter germinated most efficiently when the GerI and GerQ GRs were stimulated by millimolar concentrations of inosine (14, 15), which is not expected to be present in spent minimal medium. Hence, the 15–20% absorbance loss measured in this case probably results from the gradual dispersion of clumps of spores in the 96-well plates over the course of the experiment. In contrast, progression to outgrowth and even cell division in *B. subtilis* and *B. megaterium* spores, respectively, suggests that spent medium probably contains nutrient sources—peptides, amino acids, carbohydrates, etc.,—raising the possibility that these, and not presumed PG fragments, are the primary triggers for germination under these conditions.

### Spore germination in the presence of peptidoglycan fragment mixtures

**Fig 3:**
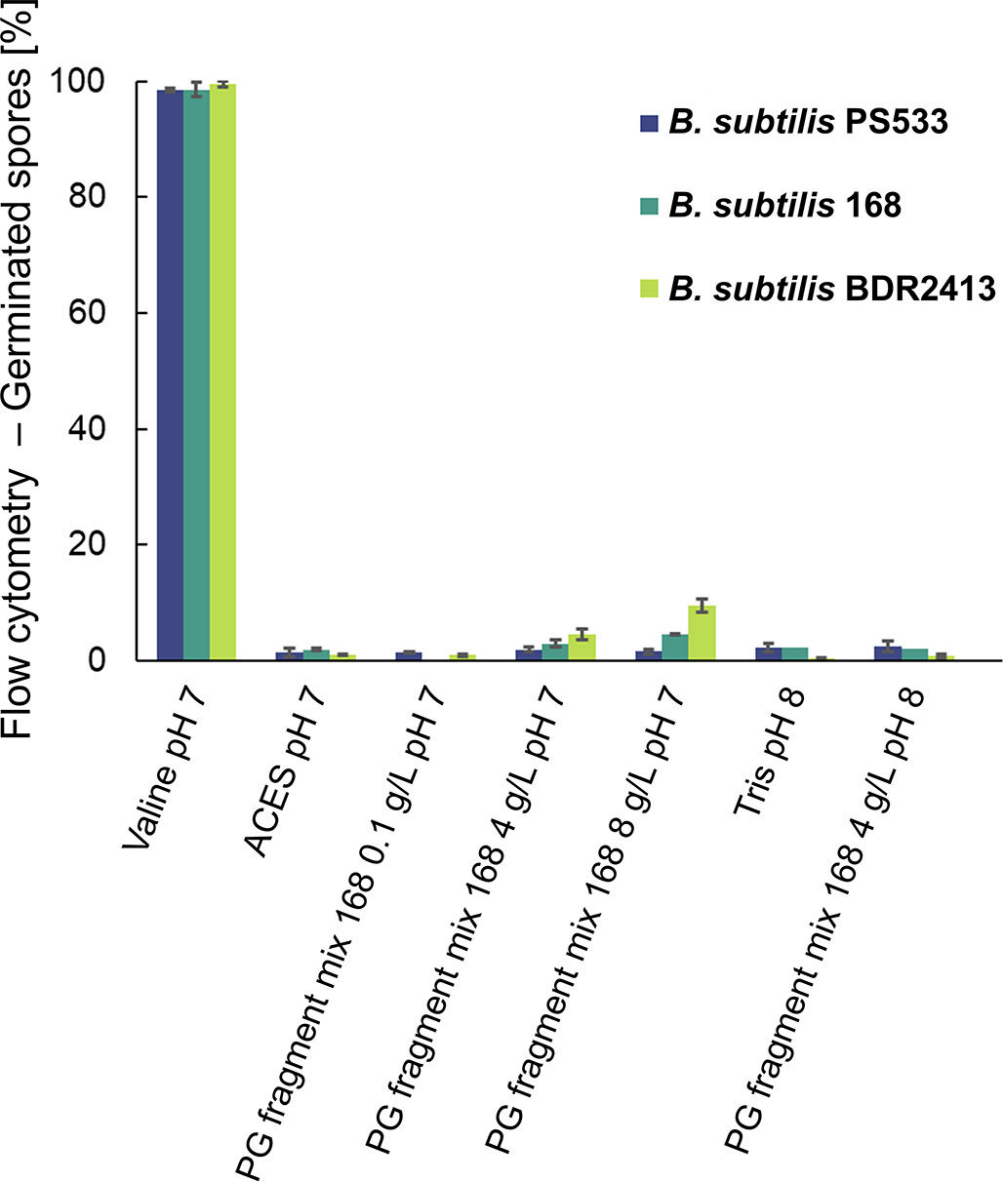
Flow cytometric quantification of germination of *B. subtilis* PS533, 168, and BDR2413 spores in the presence of the mutanolysin-derived PG fragment mixture from *B. subtilis* 168. Spores were incubated at 37°C for 3 h in 50 mM ACES buffer at pH 7 at different PG fragment concentrations or in 75 mM Tris-HCl, pH 8. Spores in buffer without PG fragments served as a negative control, whereas spores in ACES buffer with 100 mM L-valine served as a positive control. Data represent mean values ± standard deviation of independent experiments (*n* = 3, except for *B. subtilis* 168 where *n* = 2 for the 8 g/L pH 7 sample, *n* = 1 for the 4 g/L pH 8 sample, and *n* = 1 for the Tris pH 8 control).

To further investigate PG fragment germination, the germinative response of *B. subtilis* spores was assessed upon exposure to PG fragment mixtures obtained from mutanolysin-digested PG sacculi. As germination in the presence of PG fragment mixtures has been reported for *B. subtilis* 168 and PS533 spores (7, 16), we tested these strains. In fact, we tested *B. subtilis* 168 strains obtained from two different sources to take into account any inter-strain differences that may result from laboratory domestication (Table 1). Spores from the various strains were incubated in buffer supplemented with PG fragment mixtures at concentrations ranging from 0.1 to 8 mg mL^−1^. This concentration range was reported to stimulate >95% spore germination in *B. subtilis* PY79 spores within 1 hour (4). Surprisingly, our analyses indicate minimal, if any, germination across the test conditions, as ascertained by flow cytometric and absorbance measurements of spore germination (Fig. 3; Fig. S2). Thus, while spores of the three test strains exposed to 100 mM valine as a GR-mediated control measure germinated efficiently (>95%), the extent of spore germination in the presence of the PG fragment mixtures was generally comparable to that detected in buffer alone (<5%). A slightly increased level of germination was observed for spores of the BDR2413 strain when incubated with the highest concentration of the PG fragment mixture (8 mg mL^−1^; Fig. 3), raising the prospect that this strain at least may be responding weakly to certain components within the PG fragment mixture solution. However, subsequent liquid chromatography-mass spectrometry (LC-MS) analyses of the PG fragment mixtures revealed the presence of free amino acids in addition to the anticipated PG fragments (Tables S1 and S2). Notably, alanine, the L isomer of which is a potent germinant of *B. subtilis* and spores of many other species, was the most abundant amino acid detected, at a concentration of ~30 µM in the PG fragment mixture stock (24 µM in the germination experiment). Unfortunately, it wasn’t possible to determine the relative abundance of the D or L isomers of alanine in the mutanolysin digests, but regardless, the presence of alanine suggests that this was probably the trigger for germination above base-line values when spores were exposed to the highest concentrations of the PG fragment mixture. The amino acids might have been released during the enzymatic PG digestion, considering that alanine is a major component of PG (Fig. 1). The presence of coat-localized alanine racemase, which catalyzes the interconversion of L- and D-alanine, may also be significant in this regard. Carbohydrate analysis also revealed the presence of nanomolar concentrations of N-acetylglucosamine (Fig. S3), although this is not a recognized germinant of *B. subtilis* spores.

**TABLE 1:** *Bacillus* species and strains used in this work

Species	Strain	Relevant phenotype or genotype*^a^*	Source (reference)
*B. subtilis*	168	*trpC2*	Alexander Mathys, ETH Zurich
	BDR2413 (168)	*trpC2*	David Rudner, Harvard Medical School
	PS533	Prototrophic lab strain, contains plasmid pUB110 (Km^r^)	Peter Setlow, UConn Health (17)
	PY79	Prototrophic lab strain	Bacillus Genetic Stock Centre
*B. cereus*	ATCC 10876	Wild type	Anne Moir, University of Sheffield
*B. megaterium*	QM B1551	Wild type	Patricia Vary, Northern Illinois University

^*a*^ Km^r^, kanamycin resistance (10 µg/mL).

**Fig 4:**
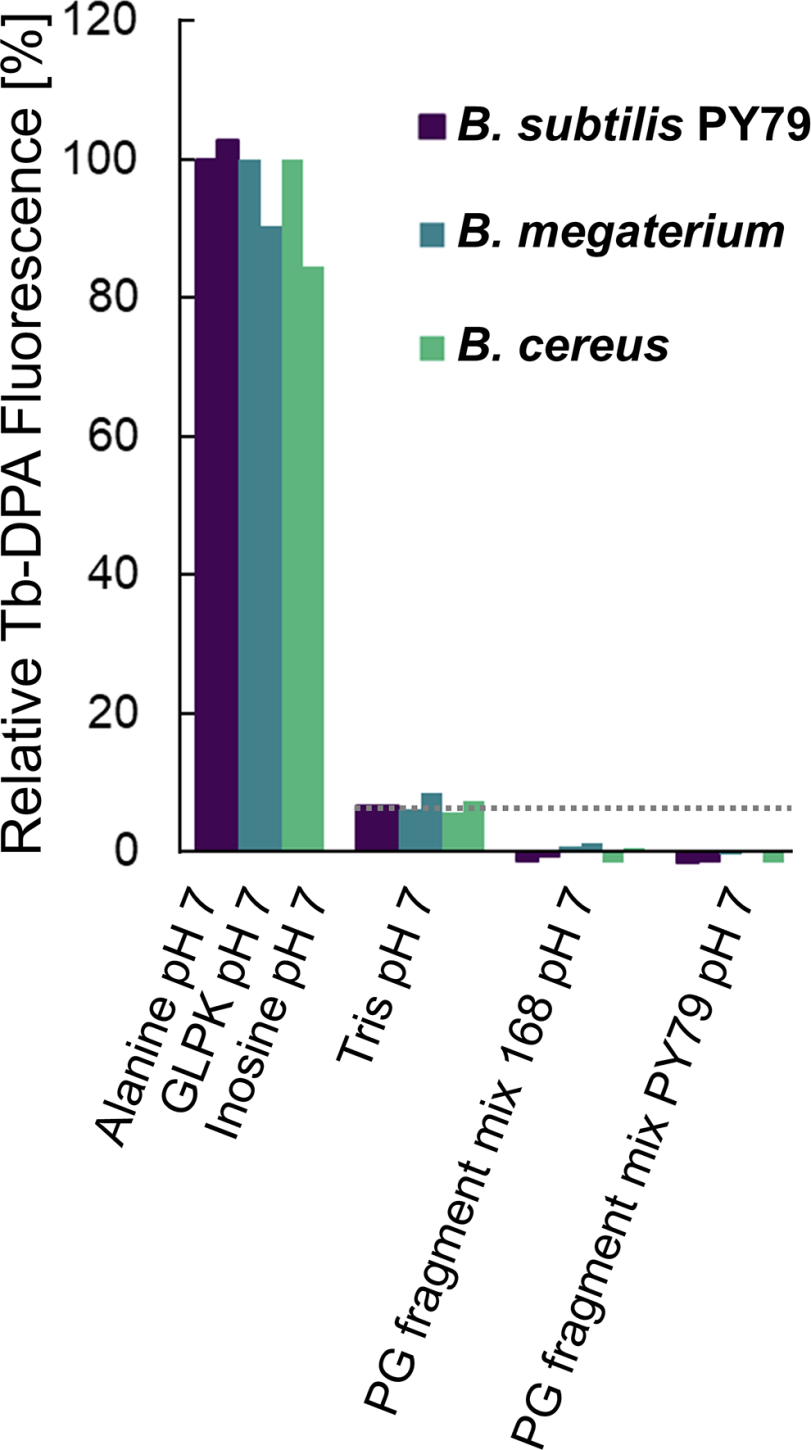
Germination of *B. subtilis*, *B. megaterium*, and *B. cereus* spores in the presence of PG fragment mixtures analyzed by Tb-DPA fluorescence assay. Spores were incubated at 37°C for 3 h in 10 mM Tris-HCl, pH 7, containing 50 µM TbCl_3_ plus 8 g/L mutanolysin-derived PG fragment mixture from *B. subtilis* 168 or PY79. Spores in buffer without PG fragments served as a negative control and indicated background fluorescence levels (- - -). Positive controls consisted of heat-shocked spores suspended in buffer supplemented with 10 mM L-alanine for *B. subtilis*, 10 mM inosine for *B. cereus*, or 10 mM each of D-glucose, L-leucine, and L-proline plus 50 mM KBr (GLPK) for *B. megaterium*. These corresponded to >90% germinated spores at endpoint as adjudged by phase contrast microscopy. Two independent experimental replicates are shown for each sample. The fluorescence level after 3 h of each species was expressed as a percentage relative to those of the first replicate of the corresponding positive control.

A similar set of experiments was conducted subsequently, this time with spores of *B. subtilis* PY79, *B. megaterium*, and *B. cereus* to test strain variability as a reason for the lack of PG fragment germination. The extent of germination was measured with the sensitive terbium-DPA fluorometric assay. In all three cases, DPA-associated fluorescence signals were below levels detected in buffer alone, indicating minimal if any spore germination in the presence of the PG fragment mixtures (Fig. 4), as confirmed subsequently by microscopy (Fig. S4). Note that similar results were obtained with the PG fragment mixtures obtained from *B. subtilis* 168 or PY79 (Table S3) and with spore incubations buffered at pH 7 and 8 (Fig. 3 and 4).

### Spore germination in the presence of purified peptidoglycan fragments

**Fig 5:**
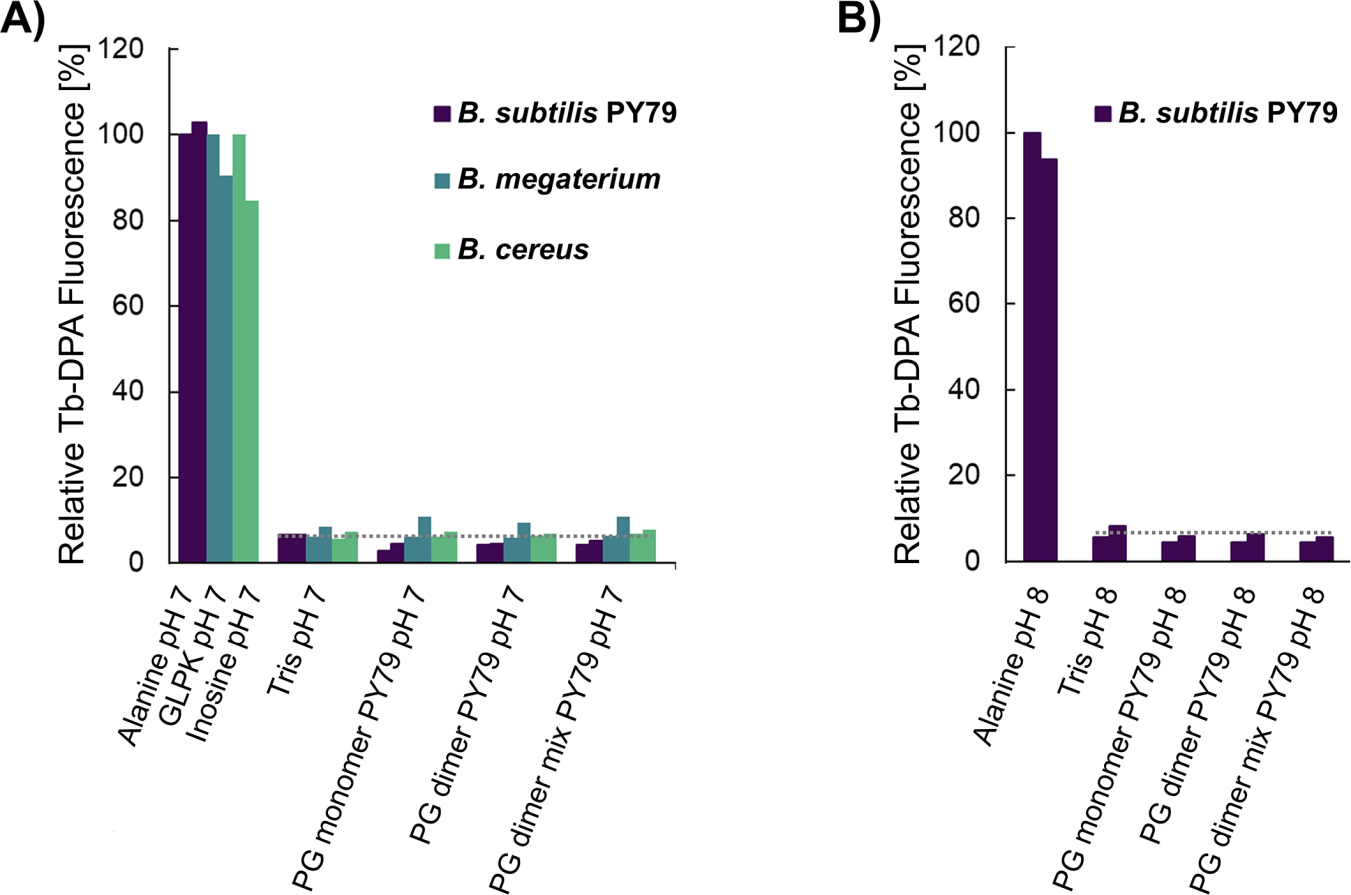
Germination of *Bacillus* spp. spores in the presence of purified PG fragments, analyzed by Tb-DPA fluorescence assay. Spores were incubated at 37°C for 3 h in (**A**) 10 mM Tris-HCl buffer at pH 7, or (**B**) in 75 mM Tris-HCl at pH 8 with 50 µM purified PG monomer, 50 µM PG dimer, or 150 µM PG dimer mixture derived from *B. subtilis* PY79. Buffers additionally contained 50 µM TbCl_3_. Spores in buffer without PG fragments served as a negative control and indicated background fluorescence levels (- - -). Positive controls consisted of heat-shocked spores suspended in buffer supplemented with 10 mM L-alanine for *B. subtilis*, 10 mM inosine for *B. cereus*, or 10 mM each of D-glucose, L-leucine, and L-proline plus 50 mM KBr (GLPK) for *B. megaterium*. These corresponded to >90% germinated spores at endpoint as adjudged by phase contrast microscopy. Two independent experimental replicates are shown for each sample. The fluorescence level after 3 h of each species was expressed as a percentage relative to those of the first replicate of the corresponding positive control.

**Fig 6:**
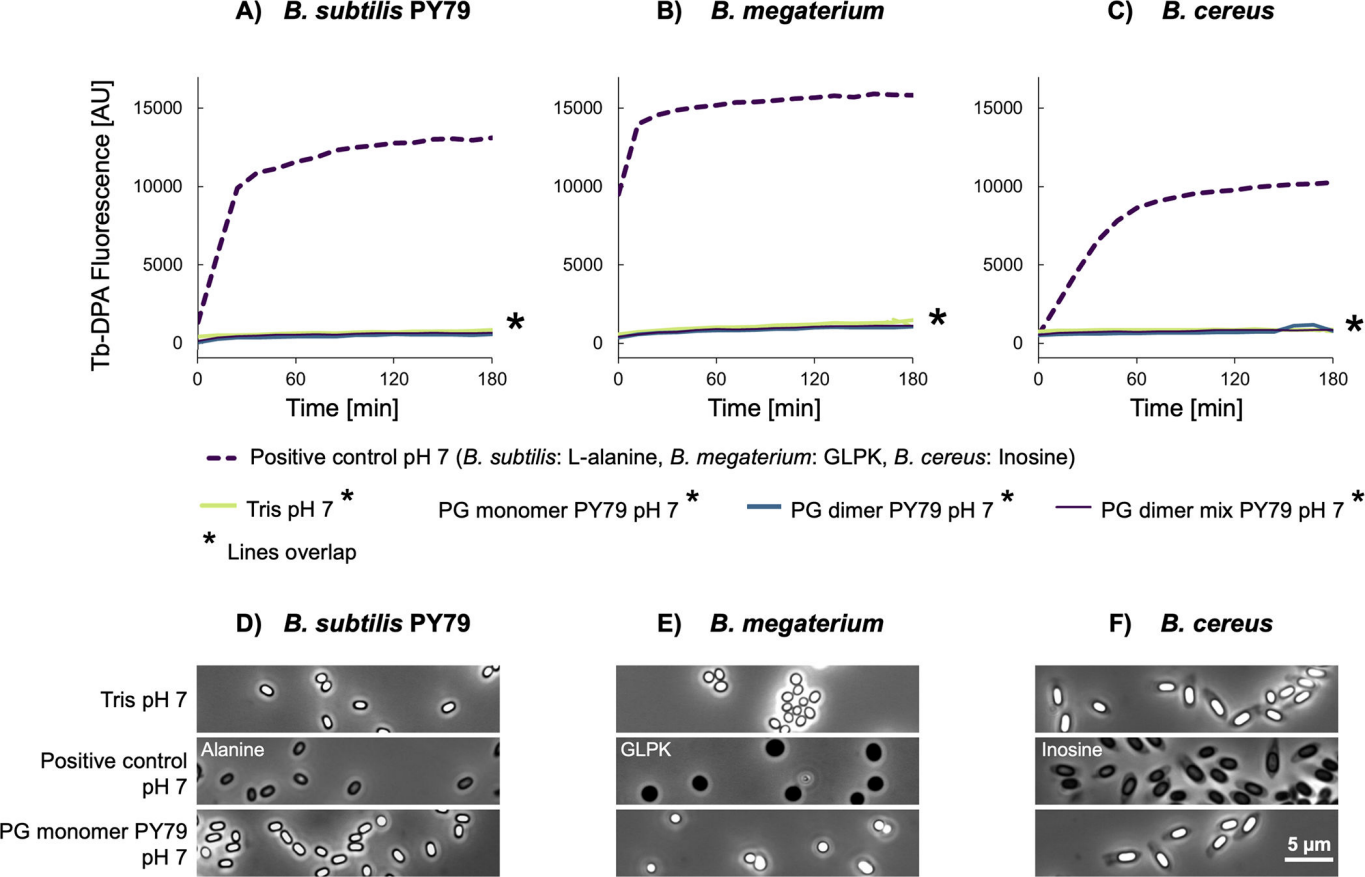
Tb-DPA fluorescence curves (**A through C**) and phase contrast microscopy images (**D through F**) of spores of *Bacillus* species after incubation in the presence of purified PG fragments. Spores were incubated at 37°C for 3 h in 10 mM Tris-HCl at pH 7 with 50 µM purified PG monomer, 50 µM PG dimer, or 150 µM PG dimer mixture from PY79. Buffers additionally contained 50 µM TbCl_3_. Spores in buffer served as a negative control. Positive controls consisted of heat-shocked spores suspended in buffer supplemented with 10 mM L-alanine for *B. subtilis*, 10 mM inosine for *B. cereus*, or 10 mM each of D-glucose, L-leucine, and L-proline plus 50 mM KBr (GLPK) for *B. megaterium*. These corresponded to >90% germinated spores at endpoint as adjudged by phase contrast microscopy. One replicate is shown for every sample. Absolute values varied between replicates, but qualitative results were similar. The last measured fluorescence values are graphed in Fig. 5.

Given the degree of uncertainty over the precise stimulus for spore germination in spent minimal medium and the lack of germinative response to the complex PG fragment mixture solution of the mutanolysin digestion (Fig. 2 to 4), we decided to assess germination in response to selected purified PG fragments. Analyses conducted in the initial PG germination study (4) indicated that muramyl disaccharide tri- or tetrapeptide moieties with *m*-Dpm at the third position of the stem peptide (Fig. 1) are required to stimulate germination. This occurs even when the muramic acid moiety is reduced to muramitol by the chemical reduction that is a prerequisite for HPLC purification. We analyzed the structures within the PG fragment mixture of *B. subtilis* PY79 by liquid chromatography with tandem mass spectrometry (LC-MS/MS) (Fig. S6; Table S3) and used this information to purify three PG fragments corresponding to the major PG monomer (gm-AEJ), the major PG dimer (gm-AEJA = gm-AEJ), and a PG dimer mixture containing the major crosslinked fragments (gm-AEJA = gm-AEJ, with varying proportions of deacetylated and amidated groups) (Fig. S8; Table S4). The fractions were quantified by NMR and used for germination assays. Spores of *B. subtilis* PY79, *B. megaterium,* and *B. cereus* were incubated in buffered solutions containing 50 µM of monomer or dimer, or 150 µM of dimer mixture, and germination was monitored by a terbium-DPA assay over a 3 hour period. In all cases, fluorescence measurements in the presence or absence of the purified PG fragments were comparable, reflecting extremely low, if any, germination (Fig. 5 and 6). This was applied to *B. subtilis* spores tested at pH 7 and 8 (Fig. 5; Fig. S10) and was confirmed subsequently for all test conditions by phase contrast microscopy (Fig. S4 and S5A).

### Spore germination in the presence of nutrient germinants and peptidoglycan fragments

**Fig 7:**
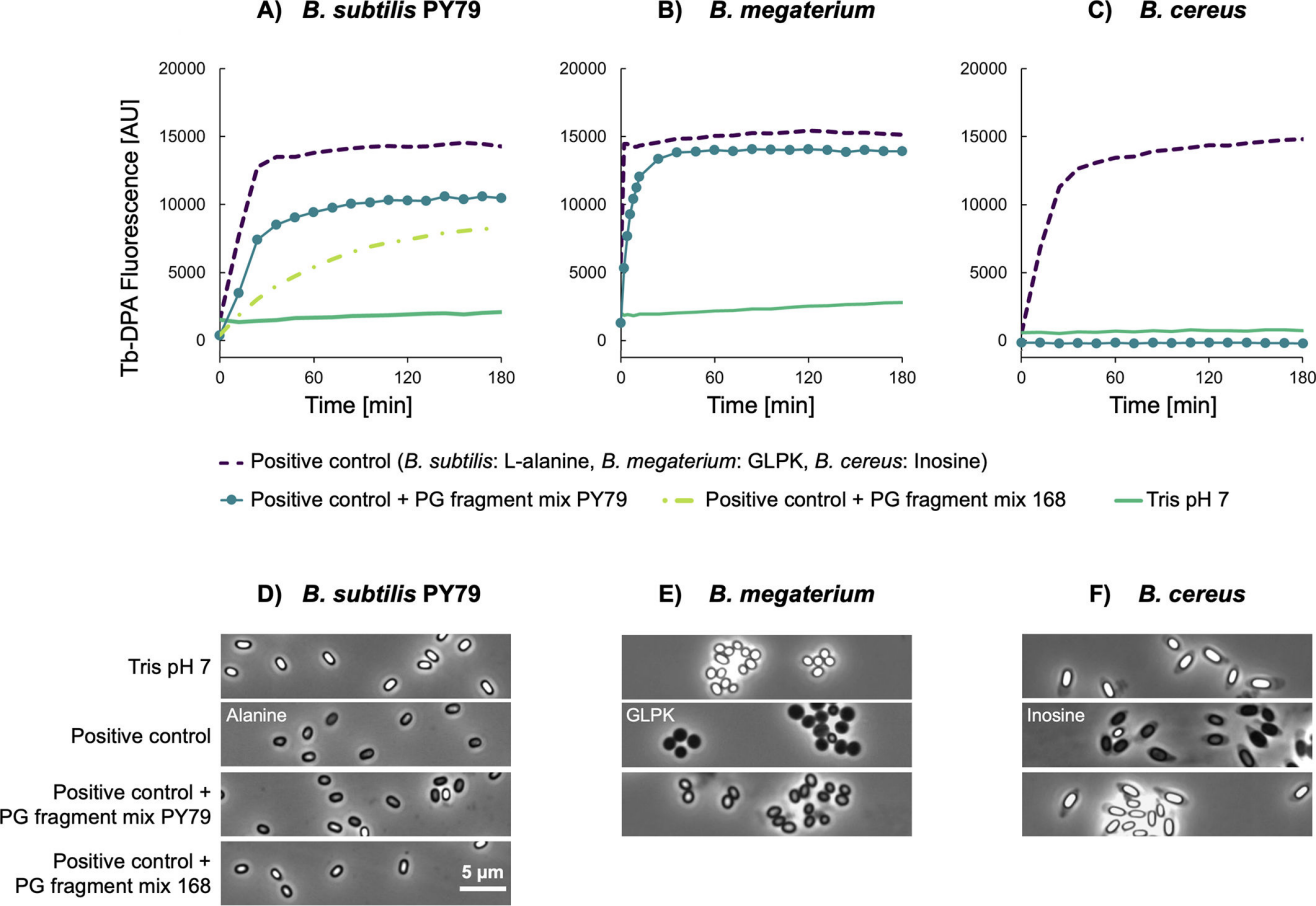
Effect of mutanolysin-derived PG fragment mixtures on GR-mediated germination. Heat-shocked *Bacillus* spores were incubated at 37°C in 10 mM Tris-HCl at pH 7 with 10 mM L-alanine for *B. subtilis*, 10 mM inosine for *B. cereus*, or 10 mM each of D-glucose, L-leucine, and L-proline plus 50 mM KBr (GLPK) for *B. megaterium*. Samples additionally supplemented with PG fragment mixtures contained 8 g/L fragments from *B. subtilis* 168 or PY79. Heat-shocked spores in buffer served as a negative control. Buffers in all cases contained 50 µM TbCl_3_. Representative individual Tb-DPA fluorescence curves (**A through C**) and phase contrast microscopy images (**D through F**) are shown.

In a penultimate set of experiments, we decided to confirm that the low germination levels with the PG fragment mixtures (Fig. 4) were attributed to a lack of germination induction by PG fragments and were not a matrix effect of the PG fragment stock solution. Therefore, we investigated whether germination could be detected after adding nutrient germinants to the PG fragment mixture. In the first instance, *B. subtilis* PY79 spores were incubated in buffer containing a saturating concentration of L-alanine supplemented with a complex PG fragment mixture. Suspensions of *B. megaterium* and *B. cereus* were prepared similarly with the appropriate nutrient germinants and the PG fragment mixture. Surprisingly, in all three cases, germination as adjudged by DPA release appeared to be diminished to varying extents in the presence of the PG fragment mixtures (Fig. 7). The effect was particularly pronounced with *B. cereus* spores, which were associated with baseline levels of DPA release and which appeared uniformly dormant despite the presence of inosine when examined by microscopy (Fig. 7F). Germination kinetics associated with *B. subtilis* PY79 spores appeared to be influenced by the source of the PG fragment mixture, with those prepared from *B. subtilis* 168 exerting an enhanced negative effect on GR-mediated germination compared to those prepared from PY79 cells (Fig. 7A and D). Detailed PG fragment analyses revealed variance in the identity and abundance of fragments present in the PG fragment mixture from both strains (Table S3), which may account for differing impacts on germination. Microscopy analyses additionally revealed that *B. subtilis* PY79 spores incubated for 3 hours with L-alanine and the *B. subtilis* 168 PG fragment mixture were distinctly more refractile than the equivalent spores incubated with the PY79-derived PG fragment mixture over the same period (Fig. 7D). *B. megaterium* spores incubated in the presence of the *B. subtilis* PY79 PG fragment mixture were similarly observed to be distinctly phase grey compared to the phase dark spores incubated with nutrient germinants alone (Fig. 7E). Despite the influence of the PG fragment mixtures on germination, the results with *B. subtilis* and *B. megaterium* spores confirm that germination initiation by nutrients could be detected in the presence of the PG fragment mixtures. Hence, the low germination signals with PG fragment mixtures alone (Fig. 4) indicate that the PG fragment mixtures did not initiate germination. The identity of component(s) present in the PG fragment mixture that appears to exert an inhibitory effect on germination has not been identified but will be investigated in future work.

**Fig 8:**
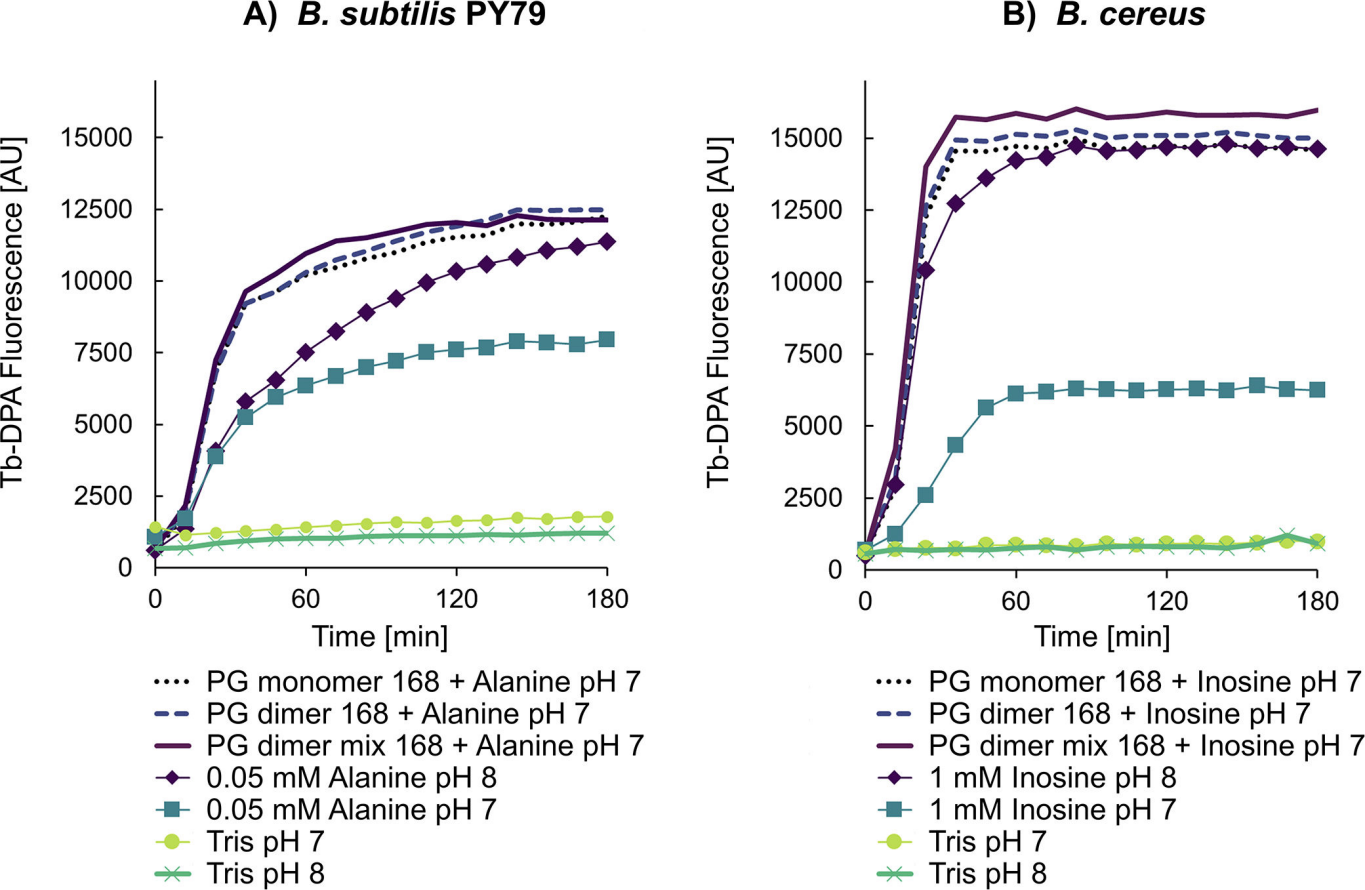
Effect of purified PG fragments on GR-mediated germination. Heat-shocked spores were incubated at 37°C in 10 mM Tris-HCl, pH 7.0, with or without (**A**) 0.05 mM L-alanine for *B. subtilis*, or (**B**) 1 mM inosine for *B. cereus* spores, and germination was measured by Tb-DPA fluorescence as described in the methods. Some samples were additionally supplemented with 50 µM purified PG monomer, 50 µM PG dimer, or 150 µM PG dimer mixture purified from *B. subtilis* 168. Heat-shocked spores in buffer served as negative controls. Buffers in all cases contained 50 µM TbCl_3_. Presented data are from a single experiment with second technical replicates shown in Fig. S11. Note that the pH of PG fragment-supplemented samples was closer to pH 7, as adjudged from pH paper.

Finally, we decided to investigate whether the presence of the purified PG fragments could influence spore germination triggered via the GRs. Hence, similar experiments were conducted with the purified PG fragments, but as a result of limited quantities, were reduced to analyses with two technical replicates with *B. subtilis* PY79 and *B. cereus* spores. Experiments were conducted under conditions where nutrient germinants were present at non-saturating concentrations in an attempt to maximize the likelihood of observing any PG fragment-mediated effects on germination. In contrast to the inhibitory effect of the PG fragment mixtures on nutrient-triggered germination, germinative rates of spores of both species were observed to increase when L-alanine or inosine was supplanted with the purified PG monomer or dimer (50 µM), or PG dimer mixture (150 µM) (Fig. 8). The observation that the purified PG fragment stocks appeared to drop the pH of 10 mM Tris buffer from pH 8 to ~7, based on analyses with pH paper, raised the possibility that the apparent positive effect on germination may be attributed to variance in the pH of the test buffers. However, control experiments conducted with nutrient germinants only at pH 7 appear to rule this out (Fig. 8), supporting the idea that certain PG fragments can enhance—but not initiate—spore germination.

## DISCUSSION

The presence of a continuous sensing mechanism designed to detect favorable changes in the host cell’s environment, while at the same time, being metabolically dormant represents one of several biological curiosities associated with bacterial spores. Research interest spanning several decades has focused on the nature of spores’ interaction with nutrient germinants and how these trigger the series of biophysical events that comprise germination. Thus, it was something of a surprise when an entirely novel and seemingly physiological route to germination was described in 2008 (4). The demonstration that *Bacillus* spp. spores could detect PG fragments infused from the surrounding medium to trigger a germinative response is consistent with the idea that these fragments betray the presence of active cells—and therefore nutrients—within the vicinity of the spore. The model would be more convincing from biochemical and physiological perspectives; however, if PG interaction with the spore led unambiguously to a signal transduction pathway commensurate with ion flux from the spore core, i.e., in a manner analogous to that associated with the nutrient GRs. Instead, the proposed PG fragment receptor is a membrane-localized Ser/Thr kinase that phosphorylates a core-localized ribosomal translocation factor (4). A rational signal transduction route to ion flux across the spore membrane from this position is difficult to comprehend given the reasons outlined in the introduction to this article.

Regardless of the conceptual challenges, two independent labs have published experimental evidence that supports PG fragment-stimulated germination of spores when PrkC is present and minimal PG-initiated responses in its absence (4, 7). Hence, the data presented in this article, which reveal essentially zero initiation of spore germination in response to PG fragment mixtures or selected purified PG fragments, are difficult to reconcile with these earlier studies.

How then might we account for such divergent data? An obvious suggestion would be that strain differences are responsible. However, we used the same PY79 strain of *B. subtilis* employed in the initial study in addition to three other laboratory strains of the same species. We also used the same minimal medium (Spizizen’s) for spent culture medium experiments, and, in some experiments, identical spore purification procedures. The latter is notable since we suspected that procedures described in the initial study (4), which have fairly harsh SDS and lysozyme steps, might be responsible for inadvertently generating damaged or aberrant spores. This proved not to be the case since spores produced by these means were fully refractile, heat resistant, and germinated normally, at least in response to L-alanine (Fig. S1). Similarly, our own results revealed the presence of alanine in the PG fragment mixtures from mutanolysin-digested PG sacculi, whereas spent minimal medium appears to be sufficiently rich to support a degree of outgrowth and cell division (although that is not to say that PG preparations in other studies necessarily contained alanine or that the chemical composition of spent medium precisely matched that used in the current study). Hence, the finger of suspicion immediately points to nutrient-mediated germination via the GRs and not via PG-stimulated PrkC. Indeed, the present study supports the idea that at least the purified PG monomer, dimer, or dimer mixture exerted positive effects on germination rates measured at the population level where germination has been initiated by established nutrient germinants. Whether this apparent cooperative response is mediated by PrkC remains to be established, but future single spore-based analyses with appropriate mutant strains should yield mechanistic insight to potential synergism between nutrient germinants and specific PG fragments. Regardless, the initial PG-germination study demonstrated that *B. subtilis* spores engineered to lack any functional GRs retain a germination response to PG fragments (4). Indeed, the kinetics of the latter are similar to those associated with GR-mediated germination, which is perhaps surprising given the proposed differences in signal transduction pathways.

Differences in the composition of PG fragment solutions used to initiate germination, whether mutanolysin-derived mixtures or purified fragments, could potentially account for the divergent germination responses being reported in the various studies. However, PG was purified from *B. subtilis* 168 or PY79 sacculi in prior studies, and in the current work, all employing similar procedures to generate and characterize the material (although we additionally purified and tested PG fragments from *B. subtilis* 168). Indeed, the PG fragment mixtures and the purified PG fragments used in the current study were characterized in terms of PG fragment and non-PG content to a level of precision that arguably extends beyond that reported in the earlier studies. This enabled the quantification of free amino acids and sugars and minor chemical modifications to PG fragments like deamidations, deacetylations, etc., conferring incremental but potentially important insights into the composition of the test material. In this context, the presence of *m*-Dpm at the third position of the stem peptide, identified previously as being a critical recognition determinant for PrkC (4), was unambiguous in the PG fragments used in the current study. However, spores that germinated normally via the GR-mediated route did not respond to *m*-Dpm-containing PG fragments delivered at concentrations that achieved full germination elsewhere (4, 7).

The current work includes an additional observation of potential significance. The apparent inhibition of GR-mediated germination in the presence of the crude PG fragment mixtures may be indicative of competitive inhibition of the spore cortex lytic enzymes. The latter is an essential component of the germination apparatus tasked with depolymerizing the spore PG cortex (2). We don’t yet have sufficient information to identify which PG fragments were responsible for the observed inhibition, nor indeed if the effect is definitively caused by a PG fragment. However, if inhibition of germination was caused by PG fragments, it could mean that certain fragments can inhibit germination, while others—such as the purified PG monomer, dimer, and dimer mixture—can promote certain germination steps. Alternatively, it is possible that other, non-PG components of the mutanolysin-digested PG solution were responsible for inhibiting germination. Given the intrinsic and applied significance of any insight into inhibition or promotion of spore germination, progress in this regard has the potential to be propitious and is worthy of further attention.

Ultimately, there are no obvious means of squaring the data presented in the present study with that published in the literature (4, 7). Therefore, until compelling evidence is produced to the contrary, it may be prudent to view the idea that spores can be *induced* to germinate by exposure to soluble PG fragments with caution.

## MATERIALS AND METHODS

### Bacterial strains and spore preparation

Bacterial strains used in this work are detailed in Table 1. *B. subtilis* strains 168, PS533, and BDR2413 were sporulated on solid Difco sporulation medium at 37°C for 3–4 days (18). The resultant spores were purified by scraping from plates and resuspending in ice-cold sterile Milli-Q water and then subject to repeat cycles of washing by centrifugation (6,000 × *g* for 10 minutes, 4°C) and resuspension in water over 7 days until adjudged to be free of cell debris and germinated spores by phase contrast microscopy. Spores of the *B. subtilis* BDR2413 strain were subject to an additional buoyant density centrifugation step to achieve the desired purity (19). *B. subtilis* PY79 and *B. cereus* 10876 cultures were sporulated on solid double-strength Schaeffer’s Glucose medium (20) and supplemented nutrient agar (21), respectively, at 30°C for 3 days before purifying the spores by repeat rounds of water washing as described above. *B. megaterium* QM B1551 was sporulated in 400 mL supplemented nutrient broth (21) at 30°C with orbital agitation (225 rpm) for 3 days and purified as above. In all cases, dormant spore purity was >97% as determined by phase contrast microscopy or flow cytometry. Spore suspensions were kept at 4°C for up to a year and washed every 2–3 weeks.

### Spent medium preparation

A single overnight colony of *B. subtilis* PY79 was cultured in 200 mL Spizizen medium (22) with vigorous agitation (225 rpm) at 37°C until a cell density (OD_600_) of approximately 1.2 was attained. The cells were sedimented by centrifugation, and the resulting supernatant was subsequently passed twice through a 0.22 µm syringe filter unit to obtain cell-free spent medium.

### Peptidoglycan fragment preparation and characterization

Preparation of PG fragments from vegetative *B. subtilis* 168 cells was adapted from (23). A single colony was used to prepare a preculture in brain heart infusion broth, which was used to inoculate 6.4 L LB Miller broth (Difco, Fisher Scientific, containing 10 g/L sodium chloride) to a starting OD_600_ of 0.03. Mid-log cells (OD_600_ = 0.7) were harvested by centrifugation (8,000 × *g* for 10 minutes at 4°C), resuspended in 20–30 mL Milli-Q water, lysed by French press (1250 PSI), and then flash frozen in liquid nitrogen before storing at −20°C for <2 weeks until further processing. The lysed cells were thawed and boiled for 30 minutes in a final volume of 96 mL containing 5% w/v SDS. Insoluble PG material was pelleted in centrifugation-stable polyallomer tubes (48,400 × *g* for 40 minutes at 20°C) and washed three times with 100 mL microwave-warmed Milli-Q water without resuspending the pellet and two times while resuspending the pellet. To remove potentially covalently bound proteins from the PG, the pellet was gently resuspended in 20 mL buffer (100 mM Tris-HCl, pH 7.4, 10 mM CaCl_2_ dihydrate, 1 M NaCl) containing 1 mg/mL pronase (Sigma Aldrich; from *Streptomyces griseus*) and incubated for 3 hours at 60°C. The cell wall pellet (19,650 × *g* for 5 minutes at 20°C) was washed with 50 mL Milli-Q water twice without resuspending the pellet and once while resuspending the pellet. The pellet was stored at −20°C overnight. To remove secondary polymers from the PG, the cell wall material was incubated in 1 M HCl (5 h, 37°C) and washed five times by centrifugation using Milli-Q water. The white pellet was frozen in liquid nitrogen and freeze-dried (−50°C, 0.1 mbar, overnight) yielding ~80 mg pure PG. To cleave the PG into fragments, PG at a concentration of 25 mg/mL was enzymatically digested by mutanolysin (1,333–1,790 IU/mL; Sigma-Aldrich, from *Streptomyces globisporus* ATCC 21553) in 20 mM NaH_2_PO_4_ dihydrate buffer (pH 5.5) with gentle shaking at 37°C overnight. Mutanolysin was heat inactivated (5 minutes in a 100°C heating block). After separating insoluble debris (10,000 × *g* for 5 minutes at 20°C), the soluble PG fragments were transferred into tubes of known weight with a perforated lid for overnight freeze-drying. The total weight of PG fragments was 111 mg, including phosphate and mutanolysin residuals. This “PG fragment mix 168” was dissolved in Milli-Q water, heated for 20 minutes at 80°C, and then cooled and stored at −20°C. Preparation of PG fragments from *B. subtilis* PY79 cells was achieved using a similar protocol, although in this case (i) bacteria were cultured as above to OD_600_ of 0.5, harvested by centrifugation, and washed with Milli-Q water and (ii) cells were not lysed using the French press, which was unnecessary since mutanolysin-derived PG fragments were intended for purification by high-performance liquid chromatography (HPLC).

The composition of PG fragment mixtures derived from chemically reduced mutanolysin digests was achieved by reverse-phase HPLC separation of PG fragments followed by mass spectrometry (Supplemental S6). Isolation and purification of selected fragments from PG fragment mixtures were achieved by collecting and freeze-drying the most abundant HPLC fractions before solubilizing them in defined volumes of Milli-Q water to generate the purified PG fragment “monomer,” “dimer,” and “dimer mix” (Supplemental S7). Procedures for determining the concentration of the purified PG fragments, free amino acids, and sugars are detailed in Supplemental sections 3, 4, and 7.

### Spore germination assays

Spore germination was monitored by recording changes in the absorbance of spore suspensions at 600 nm (OD_600_) using a Tecan Infinite-200 series plate reader. Absorbance assays were conducted by adding 20 µL of spores suspended in water (~10^9^ spores/mL) to 180 µL of buffer or spent medium to give an OD_600_ of 0.4 in a clear 96-well plate. Absorbance values were recorded every 2 minutes over a 3 hour period at 37°C with 45 second orbital shaking prior to each measurement. Raw data were corrected using equivalent spore-free samples. Unless noted, samples were tested in technical triplicate using two independent spore preparations. Spores were subject to standard heat shock procedures (80°C for 20 minutes for *B. subtilis*, 75°C for 30 minutes for *B. cereus*, and 60°C for 10 minutes for *B. megaterium*) followed by cooling on ice in cases where germination was being tested via the GR route. Samples for microscopy analyses were concentrated by centrifugation (4,000 × *g* for 5 min) then dispensed onto poly-L-lysine coated glass microscope slides and imaged using phase contrast optics on an Olympus BX53 microscope. Dormant or germinated spores appear phase-bright or phase-dark, respectively.

Spore germination was additionally monitored using a terbium-DPA fluorescence assay (24). Spores were suspended to a final OD_600_ of 1.0 in black 96-well plates in 100 µL buffer with or without nutrient germinants or PG fragments, as described in the figure captions. The buffer contained 50 µM freshly added TbCl_3_ (Sigma Aldrich). DPA release was measured by monitoring fluorescence emission at 620 nm following excitation at 337 nm for 3 hours at 37°C using a BMG Labtech CLARIOstar Microplate Reader operating in homogeneous time-resolved fluorescence mode with orbital shaking. Raw data were corrected using equivalent spore-free samples. Samples were analyzed on different days in duplicate or in triplicate where limited quantities of PG fragments permitted it.

Flow cytometry analyses were conducted using spores (10^7^–10^8^ CFU/mL, non-heat shocked) that had been incubated at 37°C for 3 hours in 50 mM ACES [N-(2-acetamido)−2-aminoethanesulfonic acid] buffer pH 7.0 supplemented with different concentrations of “PG fragment mix 168.” Similar experiments conducted at pH 8.0 used 75 mM Tris-HCl instead of ACES buffer. Spore-containing buffers that either lacked PG fragments or contained 100 mM L-valine served as negative and positive controls, respectively. Dilute NaH_2_PO_4_ dihydrate (8 mM, pH 5.5) buffer was also added to the controls since PG fragment mixtures contained NaH_2_PO_4_ from mutanolysin digest procedures. Samples intended for flow cytometry analysis were analyzed on the same day as germination experiments or were stored at −20°C for <1 week until analysis. Aliquots of spores (200 µL) were stained in dark conditions by adding SYTO16 (Molecular Probes, The Netherlands) to a final concentration of 1.5 µM. Samples were incubated at room temperature for 12 minutes and then propidium iodide (PI; MP Biomedicals, USA) was added to a final concentration of 1.5 µM with incubation continuing for a further 4 minutes. Light scattering and fluorescence associated with stained spores were measured in an LSRFortessa II flow cytometer (BD Biosciences; 1,500–15,000 events/s, 15,000 spore events recorded). High SYTO16 fluorescence is associated with germinated spores, whereas high PI fluorescence is indicative of spores with membrane damage; low SYTO16 and low PI levels are associated with dormant spores (25). Optical settings and data processing procedures are detailed in Fig. S12 and S13. Germination was assayed on three different days, and samples were analyzed on the same day (kept at 4°C) or within a week (storage at −20°C).

## Data Availability

Raw data underlying the figures and table in this study are available via ETH Zurich Research Collection [https://doi.org/10.3929/ethz-b-000709619].

## SUPPLEMENTAL MATERIAL

Supplemental figures and tables jb.00146-25-s0001.pdf Fig. S1 to S13 and Tables S1 to S7.

ASM does not own the copyrights to Supplemental Material that may be linked to, or accessed through, an article. The authors have granted ASM a non-exclusive, world-wide license to publish the Supplemental Material files. Please contact the corresponding author directly for reuse.
