# Supplementary material for: Revisiting bacterial spore germination in the presence of peptidoglycan fragments
Source: J Bacteriol. 2025 Jul 3;207(7):e00146-25. doi: 10.1128/jb.00146-25 (PMC12288470; doi:10.1128/jb.00146-25)
Supplement: Supplemental figures and tables — Fig. S1 to S13 and Tables S1 to S7. [file jb.00146-25-s0001.pdf]

Supplemental Material

Table of contents

1 Effect of spent medium on terbium – dipicolinic acid fluorescence 1

2 Analysis of PG fragment germination by absorbance measurements 3

3 Free amino acid quantification in the peptidoglycan fragment mixture 5

4 Free sugar quantification in the peptidoglycan fragment mixture 8

5 Microscopy analysis of spores in various germination test conditions 11

6 Analysis of peptidoglycan fragment structures 12

7 Isolation and quantification of purified peptidoglycan fragments 16

8 Terbium - dipicolinic acid fluorescence curves for pH 8 21

9 Effect of purified peptidoglycan fragments on spore germination triggered by nutrient germinants 22

10 Flow cytometry settings, data processing, and controls 23

References 24

1 Effect of spent medium on terbium – dipicolinic acid fluorescence

Method. For germination analysis by the Tb-DPA fluorescence assay, *B. subtilis* PY79 spores were washed repeatedly with water and were subjected to a lysozyme and SDS purification treatment to remove germinated or vegetative cells, as described elsewhere (1). Briefly, the spores were

incubated in 1 mg/mL lysozyme (Sigma Aldrich) for 1 h at 37°C, and 2% w/v SDS for 20 min at 37°C before being washed three times in Milli-Q water. After a heat shock (80°C, 20 min), the spores (20 μL) were added to 180 μL of spent medium, spent medium with nutrients, or germination buffer, to a final OD_{600} of 1.0. Each solution contained 50 μM $TbCl_3$. Spent medium was prepared as described in the main manuscript Methods section. Samples were analysed, in technical triplicates, as described in the main manuscript Methods section.

Results. Germination of *B. subtilis* PY79 spores in response to spent medium was initially tested via the fluorometric DPA release assay, comparing spores purified with and without lysozyme and SDS treatment (Fig. S1). The intention was to assess whether purification methods influence spore germination in response to spent medium. Fluorescence signals remained low for spores in spent medium but showed lower baseline fluorescence compared to those of the negative control, i.e., spores in Tris buffer. Adding 10 mM L-alanine to spent medium showed an observable increase in fluorescence but not to the levels attained in the positive control sample, indicating this method was sub-optimal for detecting germination in the presence of spent medium. The apparent suppression of fluorescence may be attributed to phosphate ions in the spent medium which can disrupt the fluorescence-causing Tb-DPA chelate (2). Accordingly, spore germination in spent medium was subsequently analysed by absorbance (main manuscript Fig. 2). Results additionally show that lysozyme and SDS treatment did not affect GR-mediated spore germination (Fig. S1).

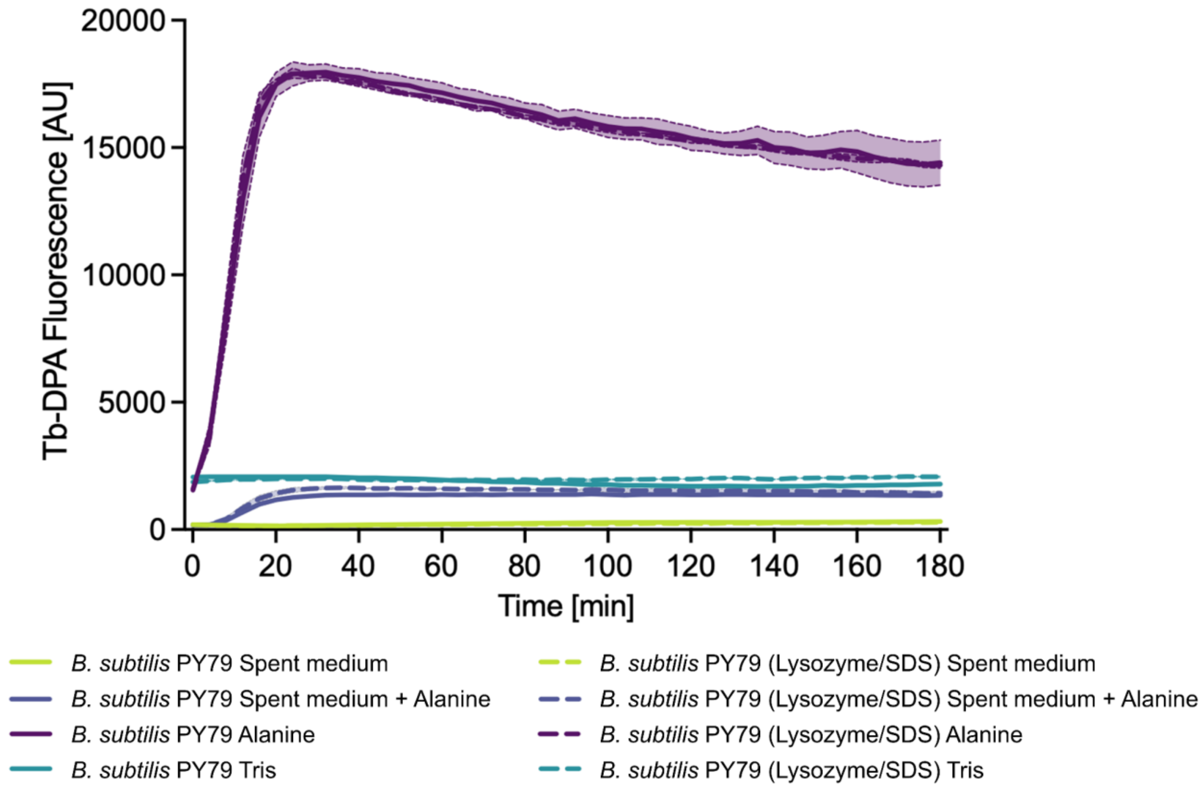

Figure S1. Germination of *B. subtilis* PY79 spores in response to spent medium, assayed by terbium-dipicolinic acid (Tb-DPA) fluorescence. Heat activated spores, with and without lysozyme and SDS treatment, were incubated at 37°C for 3 h in spent medium or spent medium supplemented with 10 mM L-alanine. Spores in 25 mM Tris-HCl pH 8.0 with and without 10 mM L-alanine served as negative and positive controls, respectively. Data is presented as the mean of technical triplicates with error envelopes representing the standard deviation.

2 Analysis of PG fragment germination by absorbance measurements

Methods. Samples were prepared as described in the main manuscript Methods section pertaining to germination in the presence of mutanolysin-derived PG fragment mixture from *B. subtilis* 168. Absorbance at 600 nm (OD_{600}) was followed in this experiment using a microplate reader (Spark, Tecan Trading AG) set with the following parameters: incubation at 37°C, continuous shaking

(360 rpm), 10 flashes per measurement, settling time of 100 ms, measurement intervals of 5 min, and total measurement duration of 180 min. The outer wells of the transparent round-bottom 96-well plate were filled with water as an evaporation barrier. One to two technical replicates (wells, 100 – 250 µL) of each spore sample or each control were analysed per day and repeated on different days. The starting OD_{600} of the spore samples was 0.4-1. The relative OD_{600} was calculated by i) subtracting the background signal ($OD_{600\_background}$) from a well of a spore-free control, i.e., with buffer and germinants, from the signal of a well with a corresponding spore sample (OD_{600_sample}) of the same volume, and ii) normalising all data points of a well to its measured OD_{600} after time (t) = 5 min ((3), Eq. 1). Relative OD_{600} values were presented as mean ± standard deviation of all replicates of a sample type for each time point.

Equation 1.

$$\text{relative } OD_{600}\ [\,] = \frac{OD_{600\_sample}(t = x) - OD_{600\_background}(t = x)}{OD_{600\_sample}(t = 5\,min) - OD_{600\_background}(t = 5\,min)}$$

Results. OD_{600} values varied considerably between single replicates of PG-supplemented samples or negative controls of the germination experiments conducted with *B. subtilis* BDR2413 (Fig. S2). In addition, the significance of the OD_{600} curves was not clear, as OD_{600} values dropped slightly for strain 168 with 8 g/L PG fragment mixture (not shown), but increased for strain BDR2413, even though very small sub-sets of spores in both samples were adjudged to have germinated by flow cytometry analyses (main manuscript Fig. 3). Ultimately, this method was deemed inappropriate for the reliable quantification of germination in PG-containing samples.

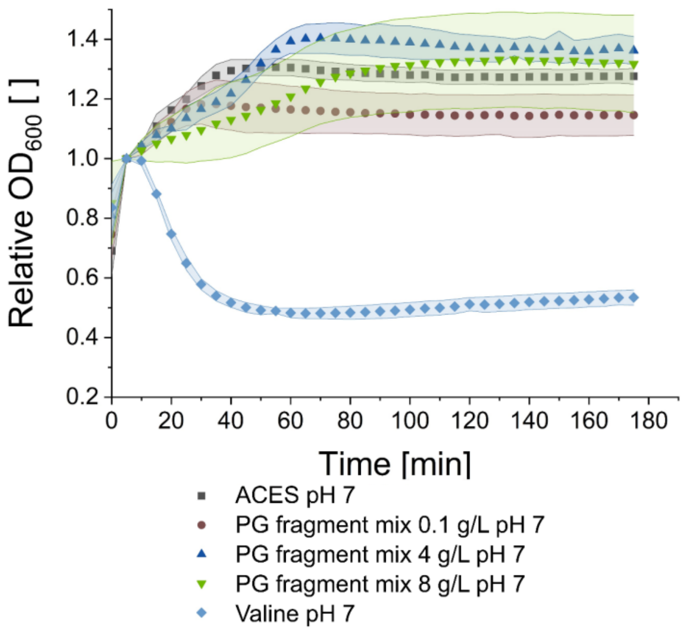

Figure S2. Germination analysis of *B. subtilis* BDR2413 (10^7-10^8 CFU/mL) in the presence of a peptidoglycan (PG) fragment mixture in 50 mM ACES buffer at pH 7 at 37°C for 3h. The PG fragment mixture was isolated derived from a mutanolysin digest of vegetative *B. subtilis* 168 sacculi. Germination is indicated by a drop of the OD at 600 nm (OD_{600}), as observed for the positive control with 100 mM L-valine as germinant and 50 mM ACES buffer. The positive and negative controls also contained 8 mM NaH_2PO_4 buffer (pH 5.5), since PG-containing samples contained NaH_2PO_4 residuals from the PG fragment mixture stock. Mean OD_{600} values from all technical replicates are shown together with corresponding error bands representing the means ± standard deviation. Technical replicates originate from 3 independent experiments with 1-2 technical replicates each (n = 3-6).

3 Free amino acid quantification in the peptidoglycan fragment mixture

Methods. The free amino acid content of the PG fragment mixture stock obtained from *B. subtilis* 168 was measured by liquid chromatography-mass spectrometry (LC-MS). Amino acids were labelled prior to the analysis based on the work by Cohen (4) and recommendations from the supplier (Waters, (5)). The standard curve per amino acid was first built by Standard H (Thermo Fisher) plus glutamic acid, glutamine, and tryptophan (Sigma) from 2.5-250 µM with the isotope-

labelled internal canonical amino acid standard 50 µM MSK-CAA-1 (Cambridge Isotope Laboratories). Labelling of standards was achieved as follows: 5 µL standard with different concentrations, 5 µL 500 µM MSK-CAA-1, 30 µL of borate buffer from the AccQ-Tag Ultra Derivatization Kit from Waters, and 10 µL 6-aminoquinolyl-N-hydroxysuccinimidyl carbamate (AQC) solution from AccQ-Tag Ultra Derivatization Kit from Waters were mixed thoroughly. The solution was incubated at 50°C for 10 min. For the sample, an aliquot of the PG fragment mixture from *B. subtilis* 168 was incubated at 37°C for 3 h before the analysis, as an experimental control to the germination treatment conditions. A volume of 100 µL of the PG fragment stock was dried via SpeedVac vacuum concentrator at 30°C and dissolved in 30 µL borate buffer (pH 9). The labelling reaction contained a mixture of 5 µL borate buffer, 5 µL 500 µM MSK-CAA-1**,** 30 µL of the PG fragment sample and 10 µL AQC. The mixture was incubated at 50°C for 10 min. After incubation, the standards and the PG fragment sample were loaded onto a Waters UPLC H-Class Plus system equipped with a Waters CORTECS® UPLC® C18 column (1.6 µm, 2.1 x 150 mm). The derivatized amino acids were detected by UV (260 nm) coupled to a Waters QDa single quadrupole mass detector in positive mode. The QDa was operated by one single MS scan, followed by selected ion monitoring (SIR) for each amino acid with a defined retention window, shown in Table S1. The column temperature was maintained at 55 °C. The injection volume was 1 µL. Gradient elution was performed using 0.1% formic acid in water as eluent A and 0.1% formic acid in acetonitrile as eluent B. The flow rate was kept constant at 0.5 mL/min with the following gradient (expressed as solvent B): Initial conditions: 1.0% B, 0.0–1min: 1% B, 1–4 min: increase to 13.0% B, 4–8.5 min: increase to 15.0% B, 8.5 -9.5 min: increase to 95.0% B, 9.5-11.5 min: 95% B, 12-15 min: decrease to 1% B. Data acquisition and data analysis were achieved using Masslynx 4.2 (Waters). Data were analysed by Targetlynx (Waters). The quantification was done by MS

trace. For standard curves, linear regression was used and the R^2 of the standard curves should reach 0.99.

Table S1. The retention time and selected ion recording (SIR) channel of the tested amino acids.

Retention time [min]	Amino acid/ Isotope-labelled standard	SIR channel [m/z]
2.05	Histidine/ 13C6 15N3 Histidine	326.2/335.2
2.40	Asparagine/ 13C6 Asparagine	303.2/309.2
2.47	Arginine/ 13C6 15N4 Arginine	345.2/355.2
2.62	Serine/ 13C3 15N Serine	276.2/280.2
2.62	Glutamine/ 13C5 15N2 Glutamine	317.2/324.2
2.72	Glycine / 13C2 15N Glycine IS	246.2/249.2
2.88	Aspartic acid/ 13C4 15N Aspartic acid	304.2/309.2
3.06	Glutamic acid/ 13C5 15N Glutamic acid	318.2/324.2
3.26	Threonine/ 13C4 15N Threonine	290.2/295.2
3.50	Alanine/ 13C3 15N Alanine	260.2/264.2
3.88	Proline/ 13C5 15N Proline	286.2/292.2
4.44	Lysine/ 13C6 15N2 Lysine	244.2/248.2 [a]
4.44	Cystine/ 13C6 15N2 Cystine	291.2/295.2 [a]
4.75	Tyrosine/ 13C9 15N Tyrosine	352.2/362.2
4.98	Methionine/ 13C5 15N Methionine	320.2/326.2
5.12	Valine/ 13C5 15N Valine	288.2/294.2
6.97	Isoleucine/ 13C6 15N Isoleucine	302.2/309.2
7.26	Leucine/ 13C6 15N Leucine	302.2/309.2
7.76	Phenylalanine/ 13C9 15N Phenylalanine	336.2/346.2
8.33	Tryptophan/ 13C11 15N2 Tryptophan	375.2/388.2

[a]*: The measured m/z of most amino acids were singly charged, except these two. They were doubly charged.*

Results. Three amino acids - alanine, glutamine, and glycine - were detected in the "PG fragment mix 168" (Table S2). The ratio of the D- to L-isoform of the amino acids could not be determined due to inhibition of the labelling reaction (6) by unknown compounds in the sample.

Table S2. Estimated concentration of free amino acids in the peptidoglycan (PG) fragment mixture from *B. subtilis* 168 (n=1).

Amino acid	Concentration [µM]
Glutamine	3.3
Glycine	0.45
Alanine	29.75

4 Free sugar quantification in the peptidoglycan fragment mixture

Methods. Free monosaccharides present in the PG fragment mixture obtained from *B. subtilis* 168 were labelled and quantified by UPLC based on monosaccharide standards. The monosaccharide standard set from AdvanceBio (formerly ProZyme) contained an equimolar quantity of 6 unlabelled monosaccharides: D-galactose, D-mannose, D-glucose, L-fucose, D-glucosamine, and D-galactosamine (GKRP-3500). This standard set for eukaryotic glycan was used for the bacterial glycan as it was readily available and sufficient to test for the presence of any free glucose or glucosamine in the PG fragment mixture stock. For labelling, the standard was dissolved in 100 μL of 0.1% formic acid resulting in a 1 nmol/μL monosaccharide standard solution. This monosaccharide solution (1 μL) was added to 5 μL 80 mg/mL sodium acetate trihydrate. The 2-aminobenzoic acid (2-AA) labelling solution was prepared by dissolving 30 mg 2AA in 1mL of 2% (w/v) boric acid in methanol, followed by addition of 30 mg sodium cyanoborohydride. A volume of 10 μL of 2-AA solution was added to monosaccharide mixtures for reaction at 80°C for 60 min. A serial dilution of the standards was prepared to obtain a standard curve. For the PG fragment mixture samples, 100 μL or 10 μL of the PG fragment mixture was dried by a SpeedVac vacuum concentrator at 30°C, dissolved each in 1 μL of 0.1% formic acid, and labelled as the standard monosaccharide solution.

A volume of 1 μL of standards or the PG fragment sample were loaded onto a Waters Acquity UPLC system with an Acquity UPLC Binary Solvent Manager and Sample Manager. Derivatized monosaccharides were detected on a Waters Fluorescence (FLR) detector (extinction: 360 nm, emission: 425 nm). The column was a Waters Acquity UPLC BEH C18 1.7 μm, 2.1 mm * 100 mm column, and the column temperature was maintained at 30°C. A gradient elution was performed

using 0.2% N-butylamine, 0.5% phosphoric acid, 1% tetrahydrofuran in water as buffer A and 50% acetonitrile as buffer B. The flow rate was kept constant at 0.294 mL/min with the following gradient (expressed as solvent B): Initial conditions: 7.0% B, 0.0–2.64 min: 7% B, 2.64–9.44 min: increase to 17.0% B, 9.44–9.82 min: increase to 100.0% B, 9.82 -13.6 min: 100.0% B, 13.6-13.97 min: decrease to 7% B, 13.97-17 min: 7% B. The data acquisition and data analysis were done by Masslynx 4.2 (Waters).

Results. D-glucosamine could be detected in the PG fragment mixture (Fig. S3: “GlcNAc”) at an estimated concentration of 0.066 or 0.065 μM for replicate 1 (10 μL sample) and 2 (100 μL sample), respectively. D-galactose, D-mannose, D-glucose, L-fucose, and D-galactosamine were below the limit of quantification, as expected, as these monosaccharides are not components of the *B. subtilis* PG preparation (7). The peak eluted at 3.85 min may be N-acetylmuramic acid based on its hydrophobicity (Fig. S3). However, the analysis service center did not have a standard to confirm the identity of this peak.

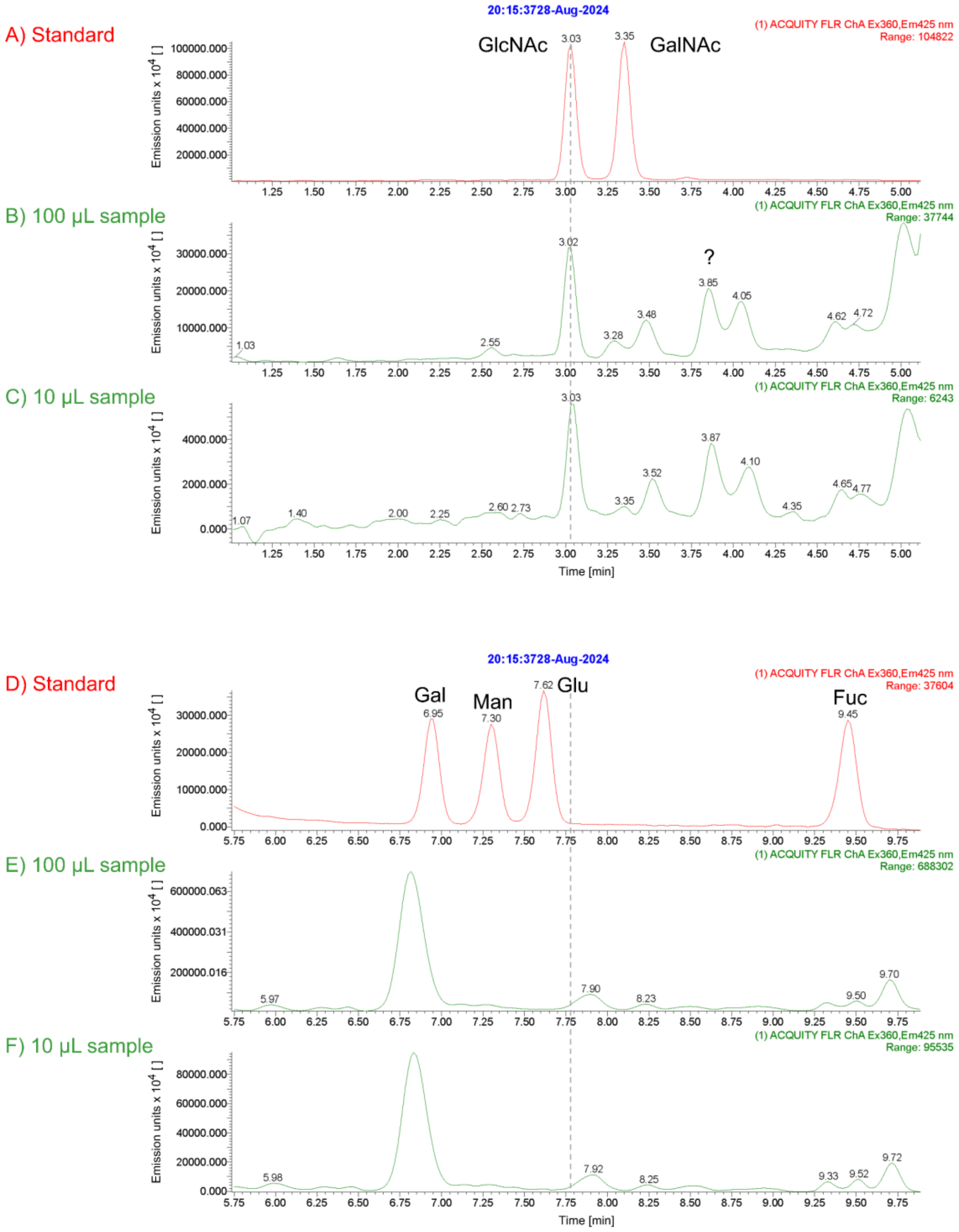

Figure S3. Chromatograms of free sugar analyses of the mutanolysin-derived peptidoglycan (PG) fragment mixture from *B. subtilis* 168. The PG fragment mixture was analysed twice using 100 µL or 10 µL PG fragment mixture (B, C, E, F) and compared to the sugar standards (A, D).

5 Microscopy analysis of spores in various germination test conditions

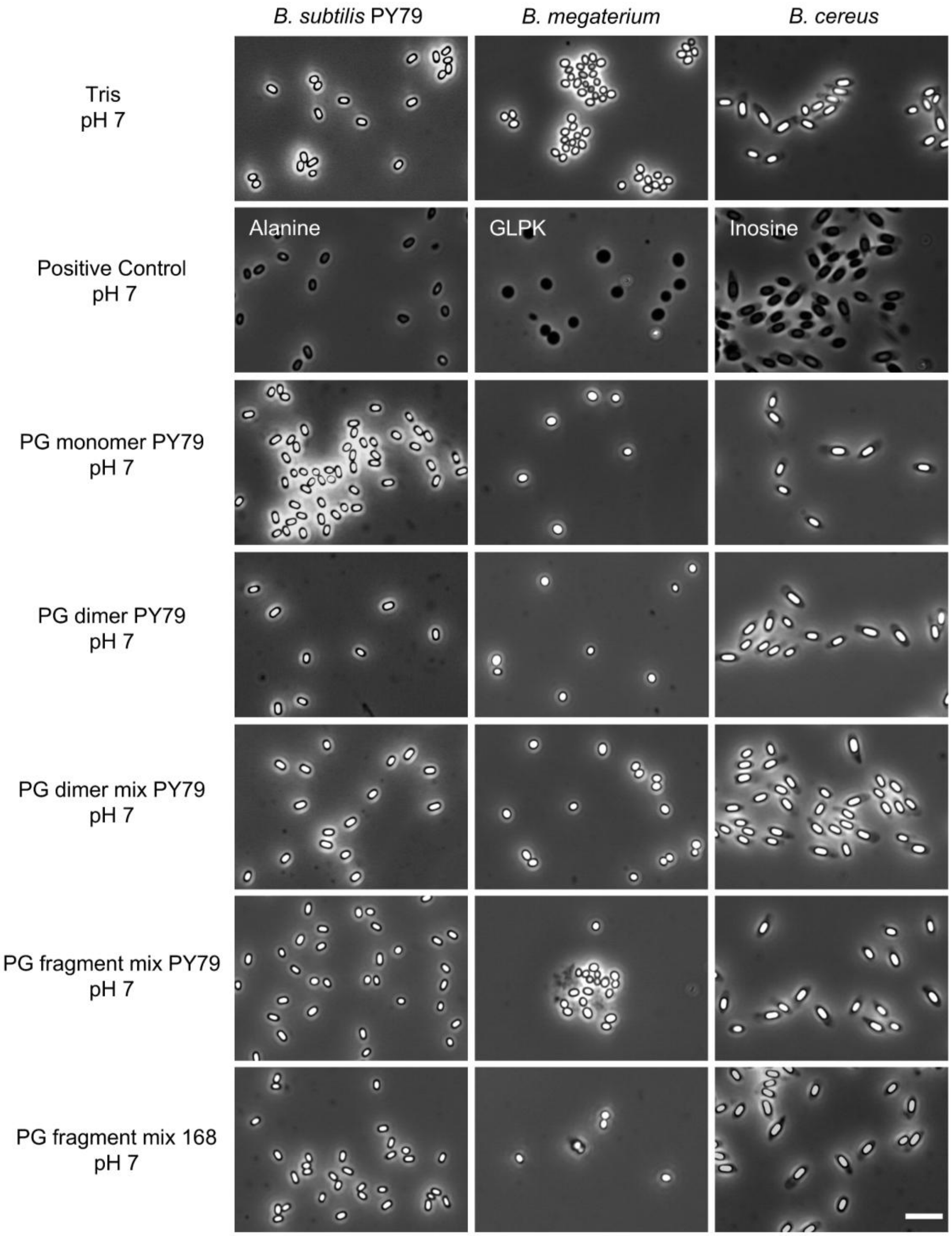

Figure S4. Representative phase contrast images of indicated spores at the end point of germination experiments described in main manuscript Fig. 3 - Fig. 6. Scale bar represents 5 μm and applies to all images.

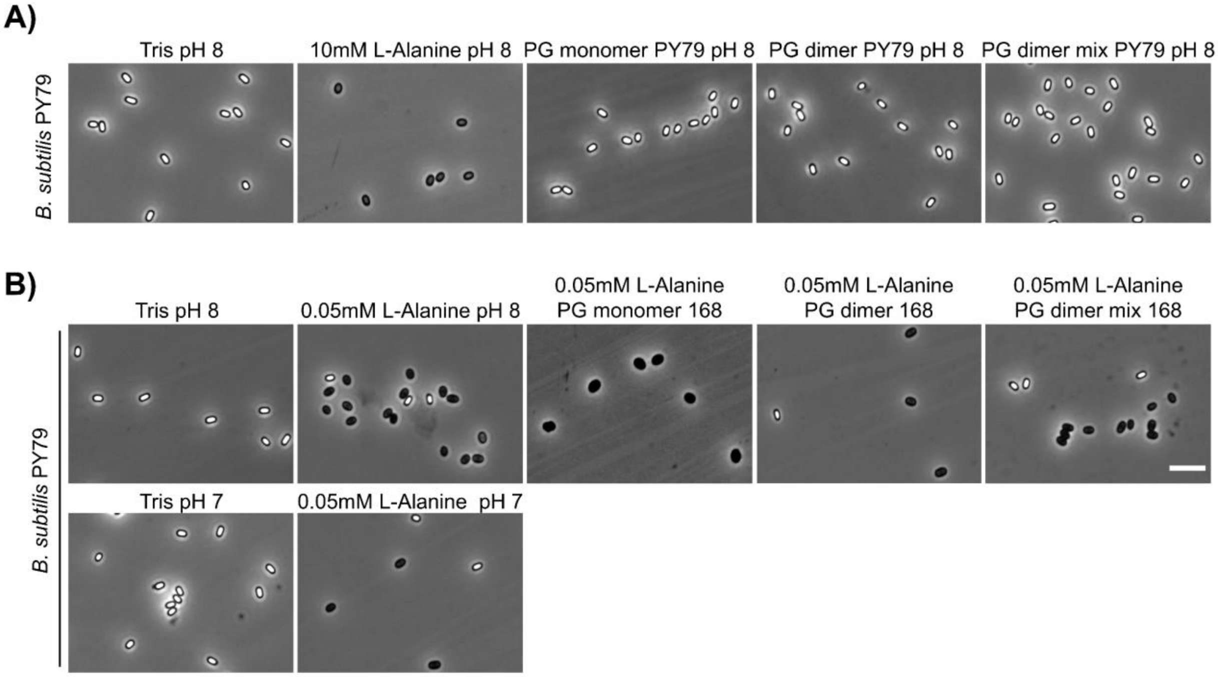

Figure S5. Representative phase contrast microscopy images of indicated spores at the endpoint of germination experiments described in Fig. 5B (A) and Fig. 8. Scale bar represents 5 μm and applies to all images.

6 Analysis of peptidoglycan fragment structures

Analysis of PG fragments was adapted from Bern et al. (8).

Method - Reduction of anomeric carbon of disaccharide. An aliquot of 250 μg of the soluble PG fragment mixtures at a concentration of 5 mg/mL was added to an equal volume of borate buffer (pH 9, 50 mL 0.25 M H_3BO_3 (CAS: 10043-35-3) + 115 mL 0.0625 M $Na_2B_4O_7 \cdot 10\ H_2O$ (CAS: 1303-96-4), filled up to 200 mL). For the reduction, 1-5% w/v sodium borohydride (CAS: 16940-66-2) was added. After incubation at room temperature for 20 min, the pH was lowered to 4.5 using 50% v/v H_3PO_4.

Method - LC-MS measurement and data analysis. Particles were removed by centrifugation (10 000 g, 5 min, 20°C). LC-MS analysis was conducted as previously described (9). In short, the samples were prepared as above and analysed using an Ultimate 3000 HPLC (Dionex/Thermo Fisher Scientific) system connected to a high resolution Q Exactive Focus mass spectrometer (Thermo Fisher Scientific). PG fragments were separated by reverse phase HPLC at 50°C using a hydrophobic C18 Hypersil Gold aQ column (150 mm length, 2.1mm inner diameter; 1.9 µm particles). Water with 0.1% v/v formic acid (buffer A) and acetonitrile with 0.1% v/v formic acid (buffer B) were used as eluents at a total flow rate of 0.3 mL/min. The buffer gradient was: 1 column volume (CV) at 100% A, 12 CV to 20% B, 3 CV at 95% B, 5 CV at 100% A. PG fragments were ionized using electrospray ionization in the positive mode. A full scan (m/z 150 -2250) was acquired at a resolution of 120 000 (full width at half maximum) at m/z 200 in the Orbitrap Exploris 240. Byos software v3.11 (Protein Metrics) served for LC-MS data deconvolution for separating overlapping signals from different analytes. PGFinder v1.0.3 software (9, 10) was used to identify PG fragments based on deconvoluted MS data. PG fragments were identified by comparison of measured masses with those of a library containing theoretical masses of abundant PG fragments (monomers, dimers, trimers) with or without the most common modifications, such as amidation, deacetylation, or anhydride. The estimated abundance of a PG fragment of a certain structure was expressed as the percentage of the fragment's ion count signal intensity in relation to the signal sum of all identified PG fragments.

Results - LC-MS analysis. Peaks in the LC-MS chromatogram (Fig. S6) and corresponding masses (Table S3) corresponded well to the expected most abundant PG fragments from vegetative *B. subtilis* 168 cells (11–13). This was also indicated by deviations of the observed and theoretical

monoisotopic masses in the expected range of < 10 ppm ((9), Table S3). Differences between the present or previous PG fragment analyses of *B. subtilis* were i) the absolute values of the PG fragment abundances, ii) the identified less abundant PG fragments, iii) the abundance of PG fragment modifications, and iv) the ratio of mono- to di-, tri, or tetramers (12, 13). These differences might be explained by several factors including different growth conditions (22, 24), the PG fragment preparation method, the LC-MS system (8), or data analysis, i.e., the PG fragment identification method.

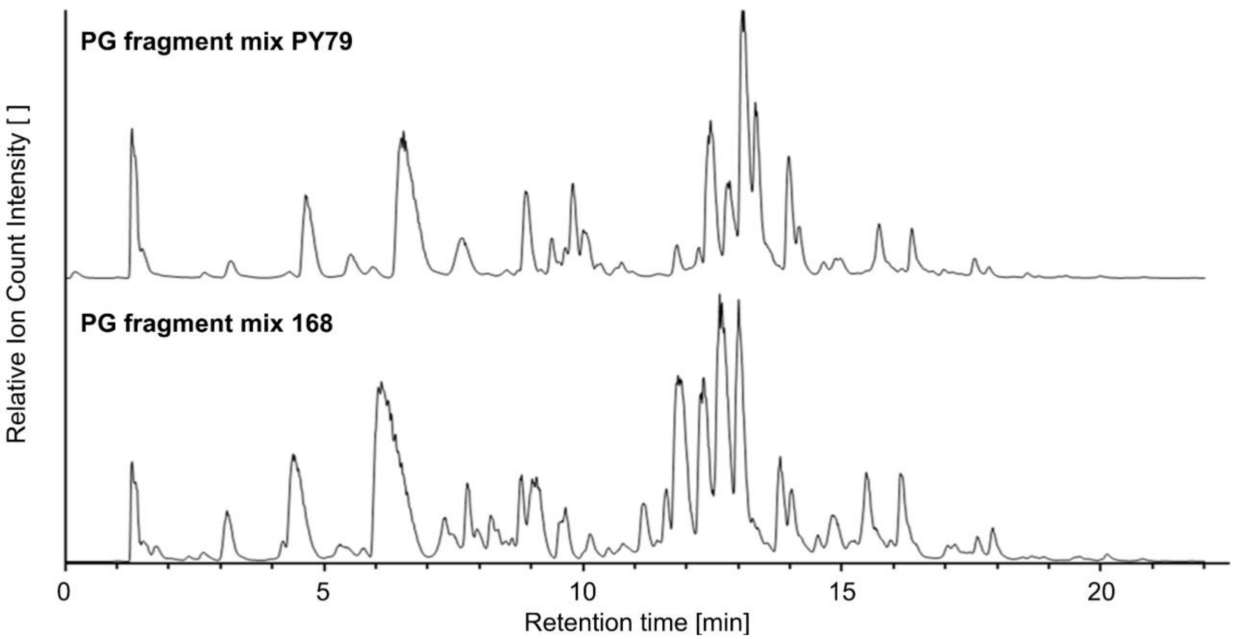

Figure S6. LC-MS chromatograms (Total Ion Count, TIC) of the peptidoglycan (PG) fragment mixture generated by mutanolysin digestion of vegetative *B. subtilis* 168 or PY79 sacculi after reduction with sodium borohydride. For each sample, the ion count intensity relative to the sample's maximal intensity is shown.

Table S3. Complete list of identified peptidoglycan (PG) fragments in the PG fragment mixtures from vegetative *Bacillus subtilis* PY79 and 168 cells depending on the retention time (RT) in the LC-MS chromatogram (Fig. S6).

	Rel. abundance [%]				
**Polymer units | PG fragment structure [a]**	**PY79**	**168**	**RT [min] [c]**	**Mass theo [Da]**	**Δppm [b]**
1 | g(-Ac)m-AEJ*	9.45%	9.67%	4.51 ± 0.13	827.376	1.3
1 | g(-Ac)m-AEJ	1.88%	1.04%	5.38 ± 0.13	828.36	0.4
1 | gm-AEJ*	11.95%	13.79%	6.30 ± 0.22	869.3866	2.2
1 | gm-AEJ	2.14%	1.99%	7.49 ± 0.18	870.3706	1.1
1 | g(-Ac)m-AEJA (Anh)	< 0.01%	ND	7.87 ± 0.00	879.3709	2.3
2 | J*A=gm-AEJ*	8.39%	5.26%	8.32 ± 0.56	1111.5245	1.1
1 | g(-Ac)m-AEJ*AG	0.05%	0.06%	8.41 ± 0.57	955.4346	0.4
1 | g(-Ac)m-AEJ* (Anh)	0.02%	0.03%	8.50 ± 0.98	807.3498	0.9
1 | g(-Ac)m-AEJ*A	0.26%	0.61%	8.76 ± 0.60	898.4131	0.5
2 | J*A=gm-AEJ	3.60%	2.81%	8.79 ± 0.60	1112.5085	0.4
1 | g(-Ac)m-AEJA	0.16%	0.17%	8.96 ± 0.58	899.3971	0.9
1 | gm-AEJ*AG	0.28%	0.24%	9.17 ± 0.56	997.4452	0.4
1 | g(-Ac)m-AEJAG	0.01%	ND	9.22 ± 0.00	956.4186	1
1 | gm-AE	10.42%	6.32%	9.30 ± 0.51	698.2858	1.5
1 | g(-Ac)m-AEJ*AA	0.07%	0.05%	9.50 ± 0.48	969.4502	0.7
1 | gm-AEJ*A	0.74%	1.06%	9.52 ± 0.49	940.4237	0.4
1 | gm-AEJA	0.36%	0.18%	9.67 ± 0.47	941.4077	0.7
1 | g(-Ac)m-AEJAA	0.01%	ND	10.29 ± 0.00	970.4343	0.7
1 | gm-AEJ*AA	0.62%	0.51%	10.43 ± 0.31	1011.4608	0.4
1 | gm-AEJAA	0.05%	0.01%	10.69 ± 0.29	1012.4448	0.9
3 | J*A=gm-AEJ*A=gm-AEJ*	0.31%	0.20%	11.90 ± 0.32	2033.9376	0.2
2 | gm-AEJ*A=g(-Ac)m-AEJ*	8.73%	8.92%	12.16 ± 0.30	1749.7891	0.5
3 | J*A=gm-AEJ*A=gm-AEJ	0.20%	0.24%	12.43 ± 0.25	2034.9216	1.1
2 | gm-AEJA=g(-Ac)m-AEJ*	4.01%	7.84%	12.56 ± 0.26	1750.7731	0.2
2 | gm-AEJ*A=gm-AEJ*	12.63%	10.54%	12.84 ± 0.23	1791.7997	0.4
1 | gm-AEJ* (Anh)	0.76%	1.29%	13.03 ± 0.28	849.3604	0.3
2 | J*A=gm-AEJ* (Anh)	0.19%	0.09%	13.10 ± 0.26	1091.4983	0.7
2 | gm-AEJA=gm-AEJ*	12.09%	13.51%	13.16 ± 0.19	1792.7837	0.1
1 | gm-AEJ (Anh)	0.15%	0.10%	13.39 ± 0.24	850.3444	0.9
2 | gm-AEJA=g(-Ac)m-AEJ	0.59%	0.90%	13.40 ± 0.15	1751.7572	0.8
2 | J*A=gm-AEJ (Anh)	0.13%	0.11%	13.89 ± 0.18	1092.4823	0.6
2 | gm-AEJ*A=gm-AEJ*A	0.34%	0.40%	13.92 ± 0.10	1862.8368	0.4
1 | gm-AEJ*A (Anh)	0.02%	0.02%	13.97 ± 0.19	920.3975	0.2
3 | J*A=gm-AEJA=gm-AEJ	0.03%	0.02%	14.03 ± 0.05	2035.9056	0.7
2 | gm-AEJA=gm-AEJ	2.01%	2.36%	14.09 ± 0.08	1793.7677	0.5
2 | gm-AEJA=gm-AEJ*A	0.30%	0.29%	14.53 ± 0.47	1863.8208	0.9
3 | gm-AEJ*A=gm-AEJ*A=g(-Ac)m-AEJ*	0.46%	0.67%	14.88 ± 0.06	2672.2023	0.6
3 | gm-AEJ*A=gm-AEJ*A=g(-Ac)m-AEJ	0.54%	1.14%	15.56 ± 0.13	2673.1863	0.3
3 | gm-AEJ*A=gm-AEJ*A=gm-AEJ*	1.31%	1.25%	15.57 ± 0.14	2714.2128	0.4
3 | gm-AEJA=gm-AEJA=g(-Ac)m-AEJ*	0.11%	0.28%	15.88 ± 0.12	2674.1703	2.0
3 | gm-AEJA=gm-AEJ*A=gm-AEJ (Anh)	0.04%	0.05%	15.88 ± 0.12	2696.1546	1.1
3 | gm-AEJ*A=gm-AEJ*A=gm-AEJ	1.47%	2.06%	16.22 ± 0.13	2715.1968	0.4
3 | J*A=gm-AEJ*A=gm-AEJ* (Anh)	0.01%	0.01%	16.45 ± 0.59	2013.9114	1.7
2 | gm-AEJ*A=g(-Ac)m-AEJ* (Anh)	0.27%	0.28%	16.51 ± 0.11	1729.7629	1.9
1 | gm-AE (Anh)	0.21%	0.18%	16.57 ± 0.17	678.2596	1.8
1 | gm-AEJ*AA (Anh)	< 0.01%	ND	16.82 ± 0.00	991.4346	2

Table continued on next page

Continued table S3					
3 | gm-AEJ*A=gm-AEJA=gm-AEJ	0.21%	0.24%	16.98 ± 0.02	2716.1808	1.1
2 | gm-AEJ*A=g(-Ac)m-AEJ (Anh)	0.14%	0.38%	17.13 ± 0.00	1730.7469	1.5
3 | gm-AEJ*A=gm-AEJ*A=gm-AEJ* (Anh)	0.13%	0.13%	17.25 ± 2.32	2694.1866	1.3
2 | gm-AEJ*A=gm-AEJ* (Anh)	1.05%	0.90%	17.57 ± 0.01	1771.7735	0.4
3 | J*A=gm-AEJ*A=gm-AEJ (Anh)	0.02%	0.02%	17.65 ± 0.04	2014.8954	0.9
2 | gm-AEJ*A=gm-AEJ (Anh)	0.78%	1.25%	17.86 ± 0.02	1772.7575	0.4
3 | gm-AEJA=gm-AEJA=gm-AEJ (Anh)	0.00%	0.00%	17.99 ± 1.45	2697.1387	4.3
2 | gm-AEJA=g(-Ac)m-AEJ (Anh)	0.02%	0.03%	18.09 ± 0.03	1731.731	2.9
3 | J*A=gm-AEJA=gm-AEJ (Anh)	0.00%	0.00%	18.53 ± 0.07	2015.8794	2.3
3 | gm-AEJ*A=gm-AEJ*A=g(-Ac)m-AEJ* (Anh)	0.03%	0.04%	18.68 ± 0.07	2652.1761	1.6
2 | gm-AEJ*A=gm-AEJ*A (Anh)	0.00%	0.00%	18.75 ± 0.05	1842.8106	1.8
2 | gm-AEJA=gm-AEJ (Anh)	0.10%	0.14%	18.84 ± 0.04	1773.7415	1.6
2 | gm-AEJA=gm-AEJ*A (Anh)	0.00%	0.00%	19.27 ± 0.29	1843.7946	1.6
3 | gm-AEJ*A=gm-AEJ*A=g(-Ac)m-AEJ (Anh)	0.04%	0.10%	19.33 ± 0.14	2653.1601	1.3
3 | gm-AEJA=gm-AEJA=g(-Ac)m-AEJ* (Anh)	0.01%	0.02%	19.81 ± 0.06	2654.1441	1.5
3 | gm-AEJ*A=gm-AEJ*A=gm-AEJ (Anh)	0.13%	0.24%	20.04 ± 0.05	2695.1706	0.4
Sum	**100%**	**100%**			

a: Polymer units: 1: monomer, 2: dimer, 3: trimer; g: *N*-acetylglucosamine, m: *N*-acetylmuramic acid, (-Ac): missing an acetyl group, (Anh): missing H_20, A: alanine, E: glutamate, J: meso-diaminopimelic acid, G: glycine, *: COOH of J amidated (=$CONH_2$).
b: The difference between observed and stated theoretical (theo) monoisotopic mass is expressed as (Δppm). [Δppm = (theoretical mass – observed mass)/theoretical mass*1 000 000], (9).
c: For a PG fragment structure > 1 RTs were possible, for example depending on the position of the amidation. The RT corresponding to the highest intensity of structure is given, while signals from all measured RTs of a PG fragment structure were consolidated for calculation of the relative abundance.
ND: not detected.

7 Isolation and quantification of purified peptidoglycan fragments

Method – Isolation of PG fragments. Individual PG fragments were isolated by HPLC using the buffers A and B as described above for LC-MS analyses of PG fragment mixtures. The buffer gradient was: 0.95 CV 100% buffer A, 9 CV to 5% buffer B, 1.8 CV at 95% buffer B, 2.4 CV at 100% buffer A. The flow rate was 1.75 mL/min. A C18 Hypersil Gold aQ column (250 mm length, 4.6 mm inner diameter; 3 µm particles) was used at 50°C. PG fragments were detected at 202 nm. The injection volume was adjusted to be as high as possible without leading to saturation of the UV detector. Collected fractions of the main peaks were kept at room temperature during the HPLC run and were frozen at -20°C until freeze-drying. The identities and purity of the isolated PG fragments were verified using the LC-MS method described above using a slightly shorter buffer gradient.

Method – PG fragment quantification. The concentration of PG fragments was determined by ^{1}H NMR by using Trimethylsilylpropanoic acid (TSP) as a standard (14). Purified PG fragments were resuspended in 10% D_2O in a final volume of 550 µL. TSP was added as a calibrant at a concentration of 364 µM (2 µL of a 100 mM stock). NMR experiments were run on a Bruker 600 MHz Neo at 20°C. Experiments were run with 16 scans and 4 dummy scans and spectra were processed using Topspin 1.4.1. Methyl singlets from N-acetyl groups expected between 1.75-2.1 ppm were used for integration. Signal intensities were integrated against the TSP peak and the final estimated concentration was determined taking sample dilution into account. The values of integral were consistent with the values from methyl doublets at ~1.4 ppm, which correspond to the alanine methyl signals present in the sample.

Results – LC-MS analysis. A representative HPLC chromatogram of PG fragment mixtures used for the isolation of the purified PG fragments is shown below (Fig. S7). LC-MS analysis of the purified PG fragment monomer, dimer, and dimer mixture from *B. subtilis* PY79 and 168 confirm their identity and high purity (Fig. S8, Table S4). While the PG fragment monomer and dimer samples from *B. subtilis* 168 or PY79 had similar composition, dimer mixtures from both strains differed in the ratio of the three most abundant PG fragments (Table S4). This aligns with different peak intensities of these PG fragments in the initial chromatogram of the PG fragment mixtures (Fig. S6, Fig. S7).

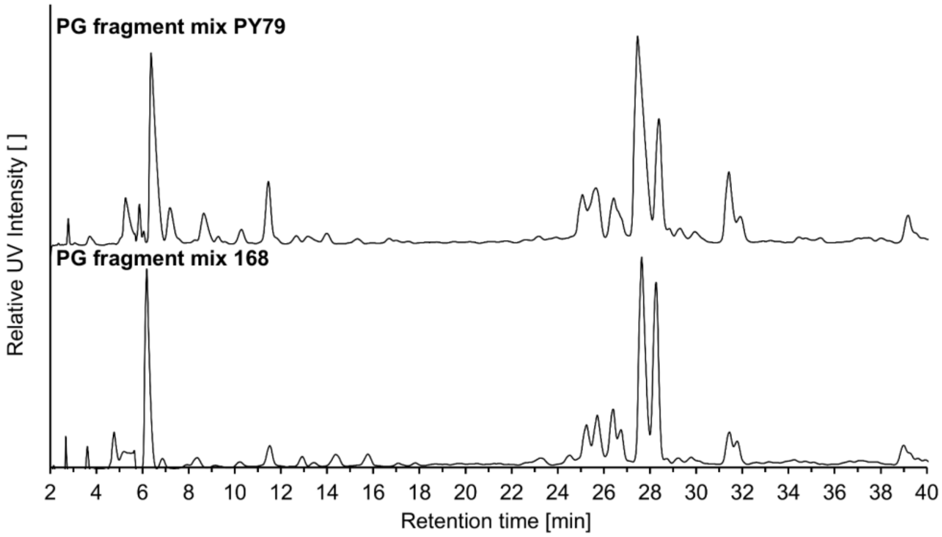

Figure S7. HPLC chromatograms (UV intensity) of the peptidoglycan (PG) fragment mixtures from vegetative *B. subtilis* 168 or PY79 cells used for the isolation of PG monomer (6.0.-6.5 min), PG dimer (28.0-28.5 min), or PG dimer mixture (25.0-28.0 min) fractions. PG fragments were reduced with sodium borohydride. For each sample, the UV intensity relative to the sample's maximal intensity is shown. Note that the peak patterns in Fig. S6 and S7 look different for the PG fragment mixtures as the detectors used were different (MS detector in Fig. S6 vs. UV detector in Fig. S7).

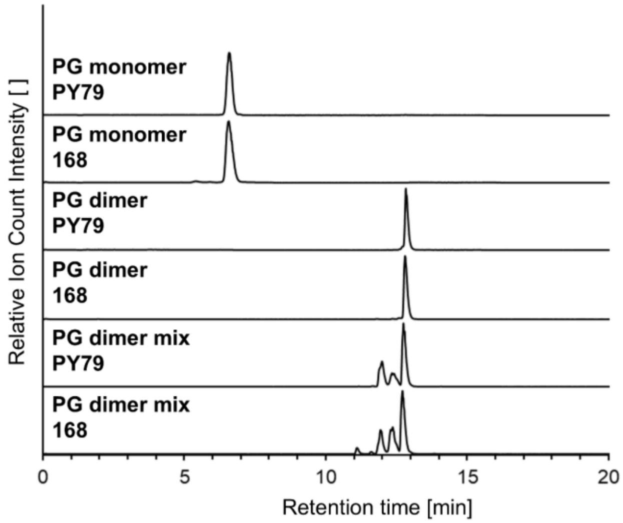

Figure S8. LC-MS chromatograms used for structure verification of the purified peptidoglycan (PG) monomer, PG dimer and PG dimer mixtures derived from vegetative cells of *B. subtilis* 168 or PY79 after PG fragment reduction with sodium borohydride. For each sample, the ion count intensity relative to the sample's maximal intensity is shown.

Table S4. Composition of purified peptidoglycan (PG) fragment fractions from *B. subtilis* 168 or PY79, corresponding to Fig. S8:

PG fragment structure	Rel. abundance [%]	RT [min]	Mass theo [Da]	Δppm [b]
***B. subtilis* 168:**				
PG monomer:				
gm-AEJ*	96.4	6.55	869.3866	-1.8
g(-Ac)m-AEJ	1.5	5.37	828.36	0.8
gm-AEJ	0.1	7.43	870.3706	0.8
J*A=gm-AEJ*	1.0	5.93	1111.5245	0.3
J*A=gm-AEJ	1.0	6.33	1112.5085	0.1
PG dimer:				
gm-AEJA=gm-AEJ*, gm-AEJ*A=gm-AEJ	97.8	12.82	1792.7837	-1.3
gm-AEJA=g(-Ac)m-AEJ*, gm-AEJ*A=g(-Ac)m-AEJ	1.0	12.71	1750.7731	0.9
gm-AEJ*A=gm-AEJ*	0.9	12.62	1791.7997	0.7
gm-AEJ*A=g(-Ac)m-AEJ*	< 0.1	12.43	1749.7891	0
J*A=gm-AEJ*A=gm-AEJ*	0.3	12.35	2033.9376	1.2
J*A=gm-AEJA=gm-AEJ	0.0	12.58	2035.9056	-0.2
PG dimer mix:				
gm-AEJ*A=gm-AEJ*	57.1	12.7	1791.7997	-2
gm-AEJA=g(-Ac)m-AEJ*, gm-AEJ*A=g(-Ac)m-AEJ	21.5	12.36	1750.7731	-1.4
gm-AEJ*A=g(-Ac)m-AEJ*	21.0	11.94	1749.7891	-1.6
gm-AEJA=gm-AEJ	0.1	11.91	1793.7677	-9.5
gm-AEJA=gm-AEJ*, gm-AEJ*A=gm-AEJ	< 0.1	13.02	1792.7837	2.9
J*A=gm-AEJA=gm-AEJ*, J*A=gm-AEJ*A=gm-AEJ	0.3	12.21	2034.9216	-0.3
J*A=gm-AEJ*A=gm-AEJ*	< 0.1	11.57	2033.9376	-0.1
***B. subtilis* PY79:**				
PG monomer:				
gm-AEJ*	99.2	6.59	869.3866	-1.3
gm-AEJ	0.4	6.96	870.3706	0.1
J*A=gm-AEJ	0.4	6.36	1112.5085	0.1
PG dimer:				
gm-AEJA=gm-AEJ*, gm-AEJ*A=gm-AEJ	94.2	12.85	1792.7837	-2.1
gm-AEJA=g(-Ac)m-AEJ*, gm-AEJ*A=g(-Ac)m-AEJ	5.0	12.74	1750.7731	-0.2
gm-AEJ*A=gm-AEJ*	0.6	12.65	1791.7997	0.2
J*A=gm-AEJ*A=gm-AEJ*	0.1	12.4	2033.9376	0.3
J*A=gm-AEJA=gm-AEJ	0.1	12.54	2035.9056	0.9
PG dimer mix:				
gm-AEJ*A=gm-AEJ*	63.7	12.75	1791.7997	-1.8
gm-AEJ*A=g(-Ac)m-AEJ*	25.8	11.99	1749.7891	-0.9
gm-AEJA=g(-Ac)m-AEJ*, gm-AEJ*A=g(-Ac)m-AEJ	9.6	12.42	1750.7731	-1.1
gm-AEJA=gm-AEJ	0.1	11.96	1793.7677	-8.8
J*A=gm-AEJ*A=gm-AEJ*	0.4	12.53	2033.9376	-0.5

Results – PG fragment quantification. A representative NMR spectrum and a summary of all quantifications is provided below (Fig. S9, Table S5).

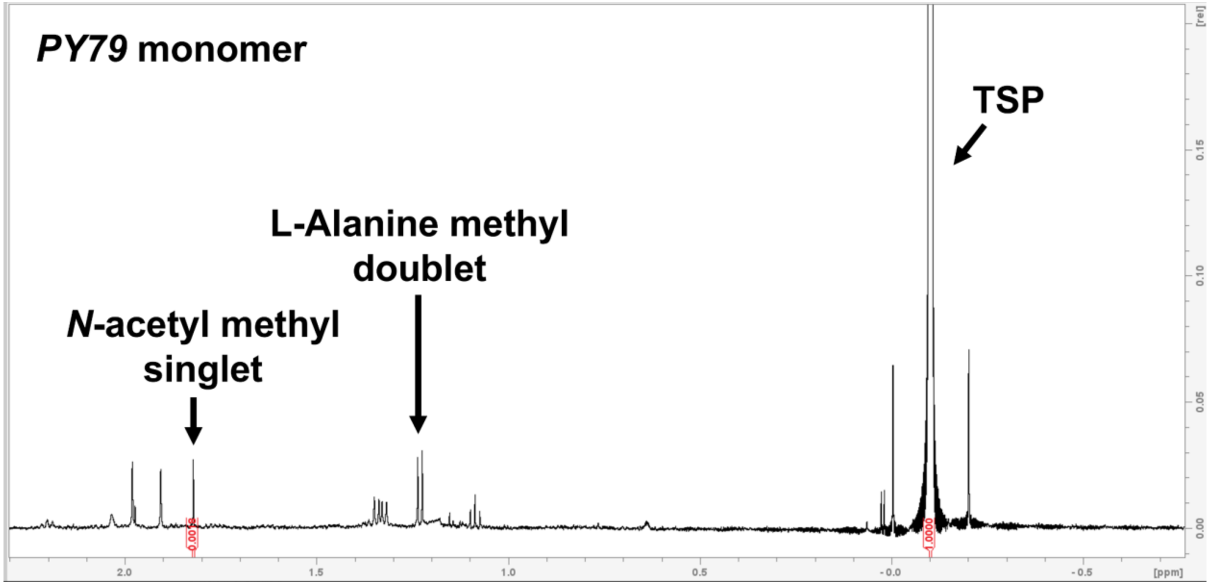

Figure S9. Exemplary ^{1}H NMR spectrum of the purified peptidoglycan (PG) monomer from *B. subtilis* PY79 compared to Trimethylsilylpropanoic acid (TSP) as a standard. The relative signal intensities depending on the chemical shift [ppm] are depicted.

Table S5. NMR quantification of isolated peptidoglycan (PG) fragments from *B. subtilis* PY79 and 168.

Sample	Sample volume	H_2O volume	D_2O volume	TSP volume	Total volume	AUC [a] *N*-acetyl	AUC [a] TSP	Concen-tration
PY79 PG monomer	10 µL	483 µL	55 µL	2 µL	550 µL	0.0018	1	110 µM
PY79 PG dimer	10 µL	483 µL	55 µL	2 µL	550 µL	0.0018	1	110 µM
168 PG monomer	10 µL	483 µL	55 µL	2 µL	550 µL	0.0020	1	120 µM
168 PG dimer	10 µL	483 µL	55 µL	2 µL	550 µL	0.0025	1	165 µM

[a] AUC: area under curve.

Note that the concentration of the PG fragments in the dimer mixture could not be determined by NMR as it contained a mixture of different chemical structures. Therefore, the concentration of the dimer mixture was estimated based on the peak area of the dimer mixture in HPLC chromatograms. The conversion factor from the peak area to concentration was established using the HPLC peak

areas of the monomer and dimer and their known concentrations. The estimated concentration for the dimer mixture from *B. subtilis* 168 and PY79 was 420 µM and 310 µM, respectively.

8 Terbium - dipicolinic acid fluorescence curves for pH 8

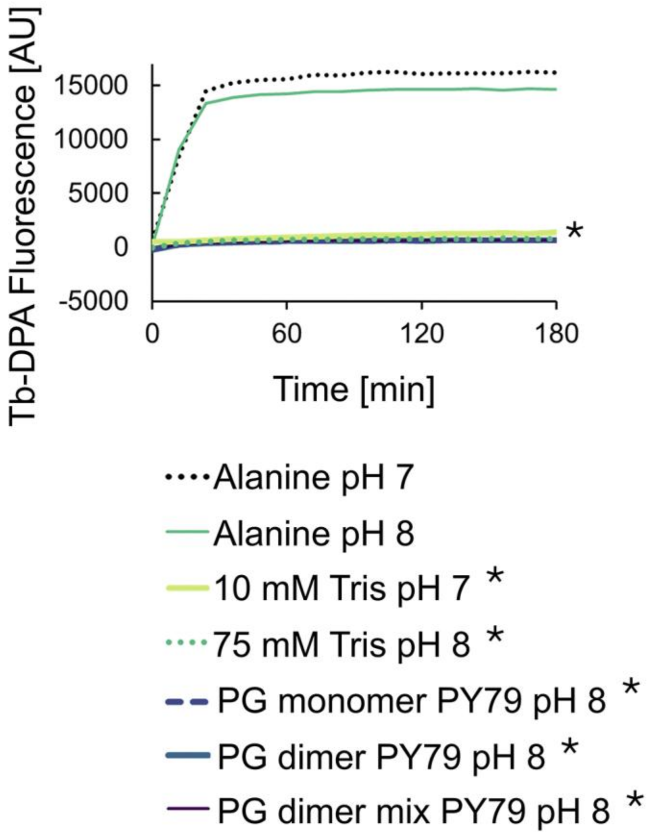

Figure S10. Terbium-dipicolinic acid (Tb-DPA) fluorescence curves of *B. subtilis* PY79 spores in the presence of purified peptidoglycan (PG) fragments. Spores were incubated at 37°C for 3 hours in 10 mM Tris-HCl at pH 7 or 75 mM Tris-HCl at pH 8 with 50 µM PG monomer, 50 µM PG dimer or 150 µM PG dimer mixture from *B. subtilis* PY79. Spores in Tris buffer served as a negative control. The positive control for germination was heat-shocked spores in buffer with 10 mM L-alanine. One replicate is shown for every sample. Absolute values varied between replicates but qualitative results were similar. The last measured fluorescence values are visualized in main manuscript Figure 5. *Lines of the negative controls and the PG-containing samples overlap.

9 Effect of purified peptidoglycan fragments on spore germination triggered by nutrient germinants

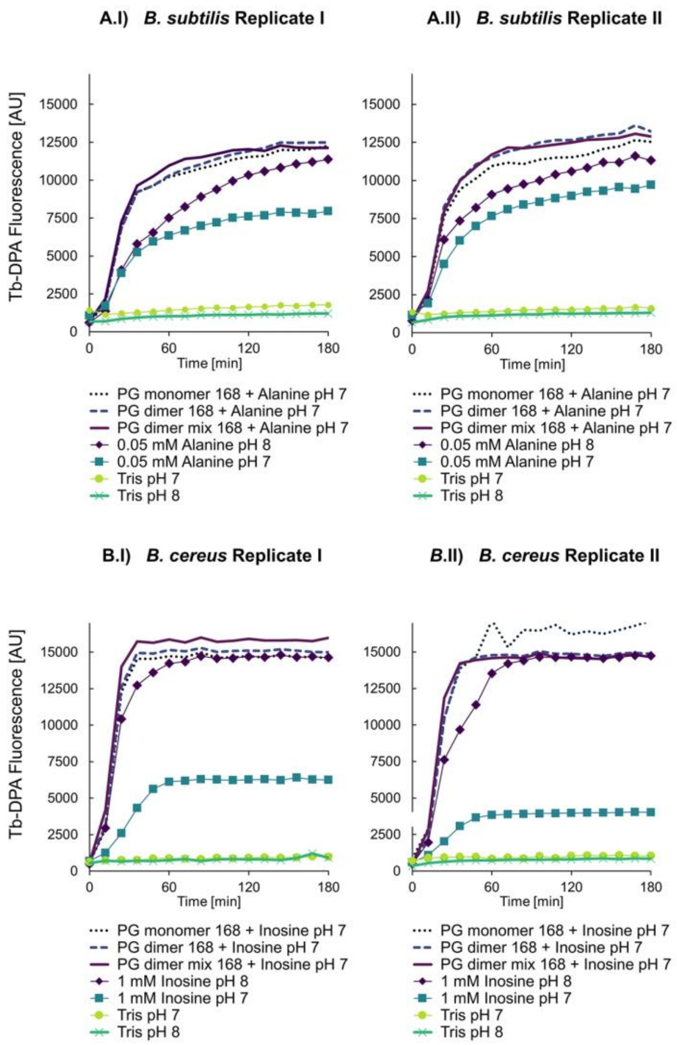

Figure S11. Effect of purified peptidoglycan (PG) fragments on GR-mediated germination. Heat-shocked spores were incubated at 37°C in 10 mM Tris-HCl, pH 7.0, with or without (A) 0.05 mM L-alanine for *B. subtilis*, or (B) 1 mM inosine for *B. cereus* spores and germination measured by Tb-DPA fluorescence as described in the methods. Some samples were additionally supplemented with 50 µM isolated PG monomer, 50 µM PG dimer or 150 µM PG dimer mix purified from *B. subtilis* 168 sacculi. Heat-shocked spores in buffer served as negative controls. Presented data represent two technical replicates. Note that the pH of PG supplemented samples was closer to pH 7, as adjudged from pH paper.

10 Flow cytometry settings, data processing, and controls

Table S7. Optical settings of the flow cytometer.

Fluorophore	Extinction	Emission
**SYTO16**	488 nm (blue laser)	530/30 nm band-pass filter (green fluorescence)
**PI**	561 nm (yellow green laser)	610/20 nm band-pass filter (red fluorescence))

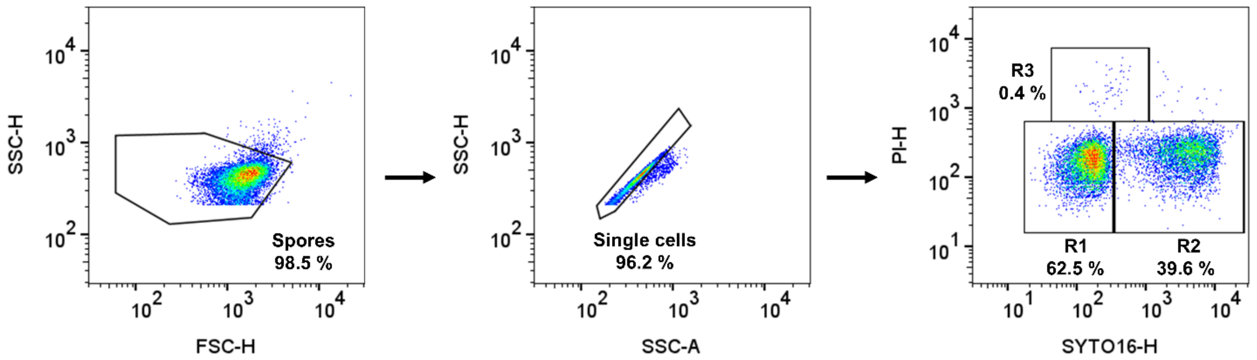

Figure S12. Gating strategy for flow cytometry measurements. Spore samples were stained with SYTO16 and PI. Events were gated for spores excluding background signals with high SSC-H values, then gated for single cells to exclude cell aggregates or multiple cells measured simultaneously, and then gated depending on the SYTO16 and PI fluorescence signals. The position of gates in SYTO16-H – PI-H plots was adapted to the absolute position of subpopulations due to variations in the PI and SYTO16 signals between samples. However, the relative gate positions were the same for each sample. Dormant spores appear in R1 and are quantified by counting the data point number in region R1 relative to the total data point number in the SYTO16-H vs. PI-H plot. R2: Germinated spores with an intact inner membrane. R3: Mostly inactivated germinated spores with membrane damage. FSC-H: forward scatter signal height, SSC-H: side scatter signal height, SSC-A: side scatter signal area, PI-H/ SYTO16-H: propidium iodide (PI)/ SYTO16 fluorescence signal height.

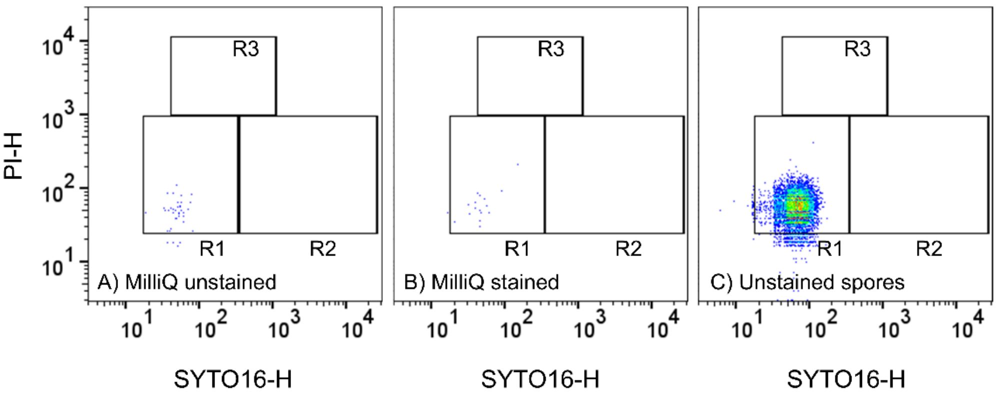

Figure S13. The following flow cytometry controls were measured on a daily basis: Filtered unstained Milli-Q water (A), and Milli-Q water stained with propidium iodide (PI) and SYTO16 (B) were recorded to monitor background signals. 'Milli-Q stained' was recorded for the same time as the spore samples; C) Unstained treated or untreated spores were measured as controls for autofluorescence. Representative plots are depicted.

References

1. Shah IM, Laaberki MH, Popham DL, Dworkin J. 2008. A eukaryotic-like Ser/Thr kinase signals bacteria to exit dormancy in response to peptidoglycan fragments. Cell 135:486–496.

2. Hagan AK, Zuchner T. 2011. Lanthanide-based time-resolved luminescence immunoassays. Anal Bioanal Chem 400:2847–2864.

3. Nagler K, Moeller R. 2015. Systematic investigation of germination responses of *Bacillus subtilis* spores in different high-salinity environments. FEMS Microbiol Ecol 91:1–10.

4. Cohen SA. 2000. Amino acid analysis using precolumn derivatization with 6-aminoquinolyl-N-hydroxysuccinimidyl carbamate, p. 39–47. *In* Amino acid analysis protocols. Springer.

5. Waters. 2024. AccQ-Tag ultra derivatization kit (web page accessed 2024-12-14). https://www.waters.com/nextgen/us/en/shop/application-kits/186003836-accq-tag-ultra-derivatization-kit.html?srsltid=AfmBOoquVTT_0N6cOQkCNg8yyKOnk0lLcAsEVcWZUq6NGhVoWB9g0HGj.

6. Yamamoto T, Yaku K, Nakagawa T. 2021. Simultaneous measurement of amino acid enantiomers in aged mouse brain samples by LC/MS/MS combined with derivatization

using Nα-(5-fluoro-2,4-dinitrophenyl)-L-leucinamide (L-FDLA). Metabolites 11:1–13.

7. Angeles DM, Scheffers DJ. 2021. The cell wall of *Bacillus subtilis*. Curr Issues Mol Biol 41:539–596.

8. Bern M, Beniston R, Mesnage S. 2017. Towards an automated analysis of bacterial peptidoglycan structure. Anal Bioanal Chem 409:551–560.

9. Galley NF, Greetham D, Alamán-Zárate MG, Williamson MP, Evans CA, Spittal WD, Buddle JE, Freeman J, Davis GL, Dickman MJ, Wilcox MH, Lovering AL, Fagan RP, Mesnage S. 2024. *Clostridioides difficile* canonical L,D-transpeptidases catalyze a novel type of peptidoglycan cross-links and are not required for beta-lactam resistance. J Biol Chem 300:105529.

10. Patel A V., Turner RD, Rifflet A, Acosta-Martin AE, Nichols A, Awad MM, Lyras D, Boneca IG, Bern M, Collins MO, Mesnage S. 2021. PGFinder, a novel analysis pipeline for the consistent, reproducible, and high-resolution structural analysis of bacterial peptidoglycans. Elife 10:1–22.

11. Liang D, Wang X, Wu X, Liao X, Chen F, Hu X. 2021. The effect of high pressure combined with moderate temperature and peptidoglycan fragments on spore inactivation. Food Res Int 148:1–8.

12. Atrih A, Bacher G, Allmaier G, Williamson MP, Foster SJ. 1999. Analysis of peptidoglycan structure from vegetative cells of *Bacillus subtilis* 168 and role of PBP 5 in peptidoglycan maturation. J Bacteriol 181:3956–3966.

13. Dajkovic A, Tesson B, Chauhan S, Courtin P, Keary R, Flores P, Marlière C, Filipe SR, Chapot-Chartier MP, Carballido-Lopez R. 2017. Hydrolysis of peptidoglycan is modulated by amidation of *meso*-diaminopimelic acid and Mg2+ in *Bacillus subtilis*. Mol Microbiol 104:972–988.

14. Gardner A, Parkes HG, Carpenter GH, So PW. 2018. Developing and standardizing a protocol for quantitative proton nuclear magnetic resonance (1 H NMR) spectroscopy of saliva. J Proteome Res 17:1521–1531.
